# Increased dosage of DYRK1A leads to congenital heart defects in a mouse model of Down syndrome

**DOI:** 10.1126/scitranslmed.add6883

**Published:** 2024-01-24

**Authors:** Eva Lana-Elola, Rifdat Aoidi, Miriam Llorian, Dorota Gibbins, Callan Buechsenschuetz, Claudio Bussi, Helen Flynn, Tegan Gilmore, Sheona Watson-Scales, Marie Haugsten Hansen, Darryl Hayward, Ok-Ryul Song, Véronique Brault, Yann Herault, Emmanuel Deau, Laurent Meijer, Ambrosius P. Snijders, Maximiliano G. Gutierrez, Elizabeth M. C. Fisher, Victor L. J. Tybulewicz

**Affiliations:** 1 The Francis Crick Institute, London, NW1 1AT, UK; 2 Université de Strasbourg, CNRS UMR7104, INSERM U1258, Institut de Génétique et de Biologie Moléculaire et Cellulaire, IGBMC, BP 10142, 1 rue Laurent Fries, 67404 Illkirch CEDEX, France; 3 Perha Pharmaceuticals, Presqu’île de Perharidy, 29680 Roscoff, France; 4 Department of Neuromuscular Diseases, UCL Institute of Neurology, London, WC1N 3BG, UK

## Abstract

Down syndrome (DS) is caused by trisomy of human chromosome 21 (Hsa21). DS is a gene dosage disorder that results in multiple phenotypes including congenital heart defects. This clinically important pathology is the result of a third copy of one or more of the approximately 230 genes on human chromosome 21, but the identity of the causative dosage-sensitive genes and hence mechanisms underlying this cardiac pathology remain unclear. Here, we show that hearts from human fetuses with DS and embryonic hearts from the Dp1Tyb mouse model of DS show reduced expression of mitochondrial respiration genes and cell proliferation genes. Using systematic genetic mapping, we determined that three copies of the *dual-specificity tyrosine phosphorylation-regulated kinase 1A* (*Dyrk1a*) gene, encoding a serine/threonine protein kinase, are associated with congenital heart disease pathology. In embryos from the Dp1Tyb DS mouse model, reducing *Dyrk1a* copy number from three to two, reversed defects in cellular proliferation and mitochondrial respiration in cardiomyocytes and rescued heart septation defects. Increased dosage of DYRK1A results in impairment of mitochondrial function and congenital heart disease pathology in mice with DS, suggesting that DYRK1A may be a useful therapeutic target for treating this common human condition.

## Introduction

Down syndrome (DS), trisomy of human chromosome 21 (Hsa21), is a common human condition resulting in many different phenotypes, including learning and memory deficits, craniofacial alterations, early-onset Alzheimer's disease and congenital heart defects (CHD) (1). DS is a gene dosage disorder with an extra copy of one or more of the genes on Hsa21 resulting in the different phenotypes. However, the identities of these causative dosage-sensitive genes remain unclear (1, 2). Discovery of such causative genes would facilitate study of the pathological mechanisms, paving the way for therapies, which are lacking for most DS clinical conditions.

With an estimated prevalence of around 1 in 800 births, DS is the most common genetic cause of CHD, with around 50% of babies with DS presenting with cardiac defects at birth (3, 4). Typically, these are ventricular and atrioventricular septal defects (VSDs and AVSDs) and malformations of the outflow tract (3–5). The most severe defects, such as AVSDs, often require surgical intervention in the first years after birth, resulting in substantial morbidity and mortality (6). Several proteins encoded by Hsa21 genes have been proposed as candidates for CHD. These include DSCAM and JAM2 adhesion molecules, COL6A1, COL6A2 and COL18A1 collagens, ADAMTS1 and ADAMTS5 metallopeptidases, the DYRK1A kinase, RCAN1, an inhibitor of calcineurin phosphatase, and SYNJ1, a regulator of endosomes (7). However, there has been no direct genetic demonstration of causality for any of these genes in studies that modulate gene dosage from 3 to 2 (2). Hence pathological mechanisms underlying this clinically important condition are poorly understood.

Hsa21 is orthologous to three regions of the mouse genome on mouse chromosome 10 (Mmu10), Mmu16 and Mmu17, with the largest of these being on Mmu16 (2). In previous work we generated the Dp1Tyb mouse model of DS that has an extra copy of the entire 23 Mb Hsa21-orthologous region of Mmu16 containing 145 coding genes in a tandem duplication, thereby genetically recapitulating trisomy of around 62% of Hsa21 genes (8). Dp1Tyb mice have a broad range of DS-like phenotypes (8, 9). Importantly, these mice have CHD similar to those seen in humans with DS, with ~50% of embryonic day 14.5 (E14.5) Dp1Tyb embryos showing VSDs, AVSDs and outflow tract defects (8). Furthermore, making use of a series of mouse strains with an extra copy of shorter segments of the Hsa21-orthologous region of Mmu16, we were able to show that an extra copy of just 39 coding genes in Dp3Tyb mice is sufficient to cause CHD, and that this region must contain at least two causative genes (8).

Here we use transcriptomics of human DS fetal hearts and mouse embryonic hearts from the DS mouse models to show that reduced expression of mitochondrial respiration genes and cell proliferation genes correlates with CHD pathology. Using systematic genetic mapping we demonstrate that one of the causative genes for CHD in DS is *Dyrk1a*. We show that increased dosage of *Dyrk1a* results in impaired cell proliferation and mitochondrial respiration of cardiomyocytes and is necessary to cause CHD in DS.

## Results

### Embryonic hearts from human DS fetuses and mouse models of DS show transcriptomic similarities

**Figure 1:**
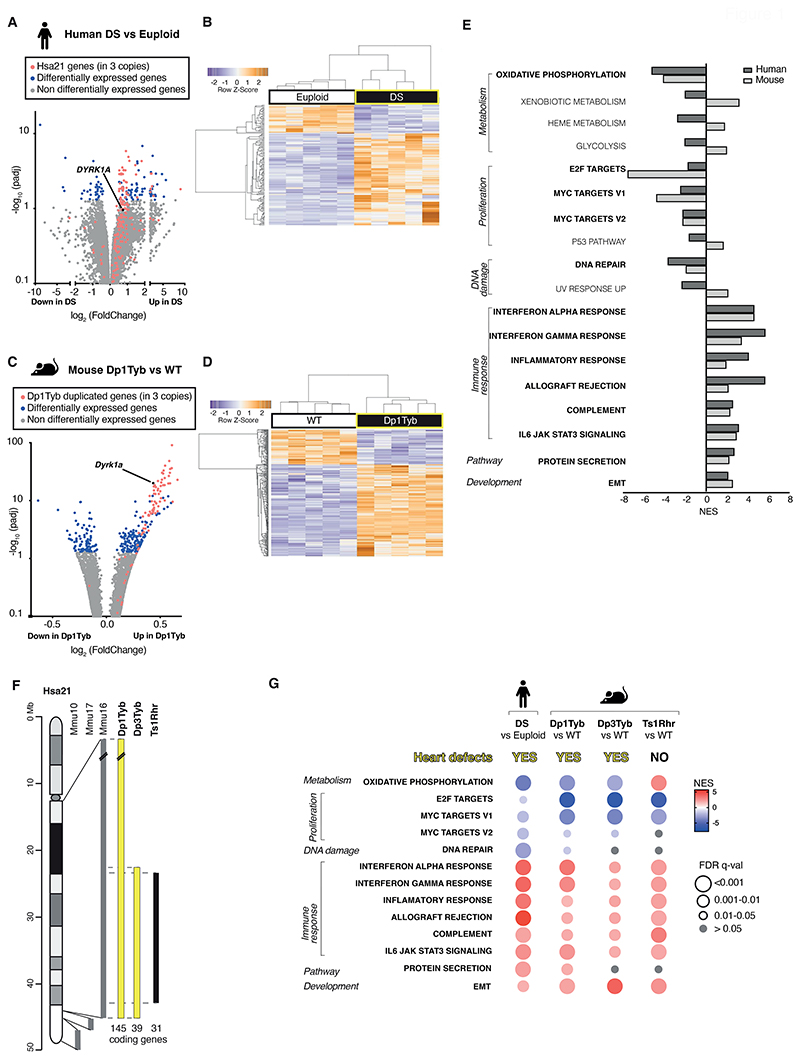
Transcriptomic similarities in embryonic hearts from human fetuses with DS and mouse models of DS. (**A, B**) RNAseq analysis of human DS and euploid embryonic hearts (13-14 post-conception weeks, *n*=5), showing (A) a volcano plot of fold-change in gene expression (DS versus euploid) against adjusted *P*-value for significance of the difference, showing Hsa21 genes (red), differentially expressed genes (blue), and *DYRK1A* (black) (B) unsupervised hierarchical clustering of the 10 samples showing heatmap of differentially expressed genes (**C, D**) RNAseq analysis of E13.5 hearts from WT and Dp1Tyb embryos (*n*=5) showing (C) a volcano plot as in A, indicating genes in 3 copies in Dp1Tyb mice (red), differentially expressed genes (blue), and *Dyrk1a* (black) and (D) hierarchical clustering of the samples as in B. (**E**) Hallmark gene sets from the Molecular Signatures Database that are significantly enriched or depleted (GSEA, ≤5% FDR) in both human DS and Dp1Tyb mouse hearts; NES, normalized enrichment scores. Gene sets showing the same direction of change in human and mouse data are indicated in bold. (**F**) Map of Hsa21 (length in Mb) showing cytogenetic bands and regions of orthology to Mmu10, Mmu17 and Mmu16 (grey) and indicating regions of Mmu16 that are duplicated in mouse strains (bold) that show CHD (yellow) and that do not (black); numbers of coding genes indicated below duplicated regions. (**G**) Comparison of dysregulated gene sets determined by GSEA of RNAseq data from human DS versus euploid embryonic hearts and in hearts from Dp1Tyb, Dp3Tyb and Ts1Rhr mouse embryos compared to WT controls. All show heart defects except Ts1Rhr mice. Colors and sizes of circles indicate NES and FDR q-value, respectively.

To discover biochemical pathways that are altered in DS and contribute to the pathology of the cardiac defects, we used RNA sequencing (RNAseq) to analyze the transcriptome of human DS fetal hearts and age- and sex-matched euploid controls (table S1). Differential gene expression analysis showed that human DS hearts had upregulated expression of many Hsa21 genes (Fig. 1A, table S2). In addition, expression of a further 99 genes was significantly altered (*P*<0.05) and hierarchical clustering demonstrated that the transcriptomes of DS and euploid human fetal hearts were distinct (Fig. 1A, B).

To investigate further, we made use of the Dp1Tyb mouse model of DS, which has CHD similar to those seen in humans with DS (8). Focusing on embryonic day 13.5 (E13.5), which is the day before heart septation is completed, RNAseq analysis of Dp1Tyb and wildtype (WT) control mouse hearts showed that the genes present in three copies in Dp1Tyb mice were increased in expression by about 1.5-fold, as expected (Fig. 1C, fig. S1A, table S3). In addition, a further 243 genes showed altered expression in Dp1Tyb embryonic hearts, and hierarchical clustering showed that Dp1Tyb and WT mouse embryonic hearts were transcriptionally distinct (Fig. 1D). Gene set expression analysis (GSEA) (10) revealed significant changes (*P*<0.05) in multiple pathways in human DS and mouse Dp1Tyb embryonic hearts (Fig. 1E). Most of these pathways were altered in the same direction in both species, including decreased oxidative phosphorylation and cellular proliferation, and increased immune responses and epithelial to mesenchymal transition (EMT) compared to control subjects, showing that the transcriptional changes in Dp1Tyb mouse embryonic hearts resembled those in human DS hearts (Fig. 1E).

### Decreased expression of oxidative phosphorylation and cell proliferation genes correlates with CHD

To identify which pathways were most likely to be involved in CHD pathogenesis, we extended the RNAseq analysis to two further mouse strains: Dp3Tyb and Ts1Rhr. Dp3Tyb embryos show CHD similar to those in Dp1Tyb mice and DS, but this strain has an extra copy of just 39 protein-coding genes. These genes are entirely contained within the large duplication in Dp1Tyb mice, thus demonstrating that these 39 genes are sufficient to cause CHD (Fig. 1F) (8). In contrast, Ts1Rhr embryos have an extra copy of a slightly shorter region containing just 31 genes and do not show CHD (8). Comparison of the transcriptional changes in Dp3Tyb and Ts1Rhr mouse embryonic hearts with those of mouse Dp1Tyb and human DS hearts, showed that the changes in DNA repair and protein secretion pathways were not seen in either Dp3Tyb or Ts1Rhr hearts and these were not considered further (Fig. 1G). Increased expression of immune response and EMT pathways was seen in both Dp3Tyb and Ts1Rhr hearts, demonstrating that these changes are not sufficient to cause CHD (Fig. 1G, tables S4 and S5). In contrast, decreased expression of oxidative phosphorylation genes correlated with CHD – it was seen in human DS fetal hearts, and in Dp1Tyb and Dp3Tyb mouse embryonic hearts, but not in Ts1Rhr mouse hearts (Fig. 1G, fig. S1B, C). Decreased expression of the 3 proliferation gene sets was seen in Dp3Tyb mouse hearts, but only 2 of these were also decreased in Ts1Rhr mouse hearts. These results suggest that impaired oxidative phosphorylation, and potentially cellular proliferation may contribute to the etiology of CHD.

### scRNAseq reveals similar gene expression changes across different cell types in Dp1Tyb mouse embryonic hearts

**Figure 2:**
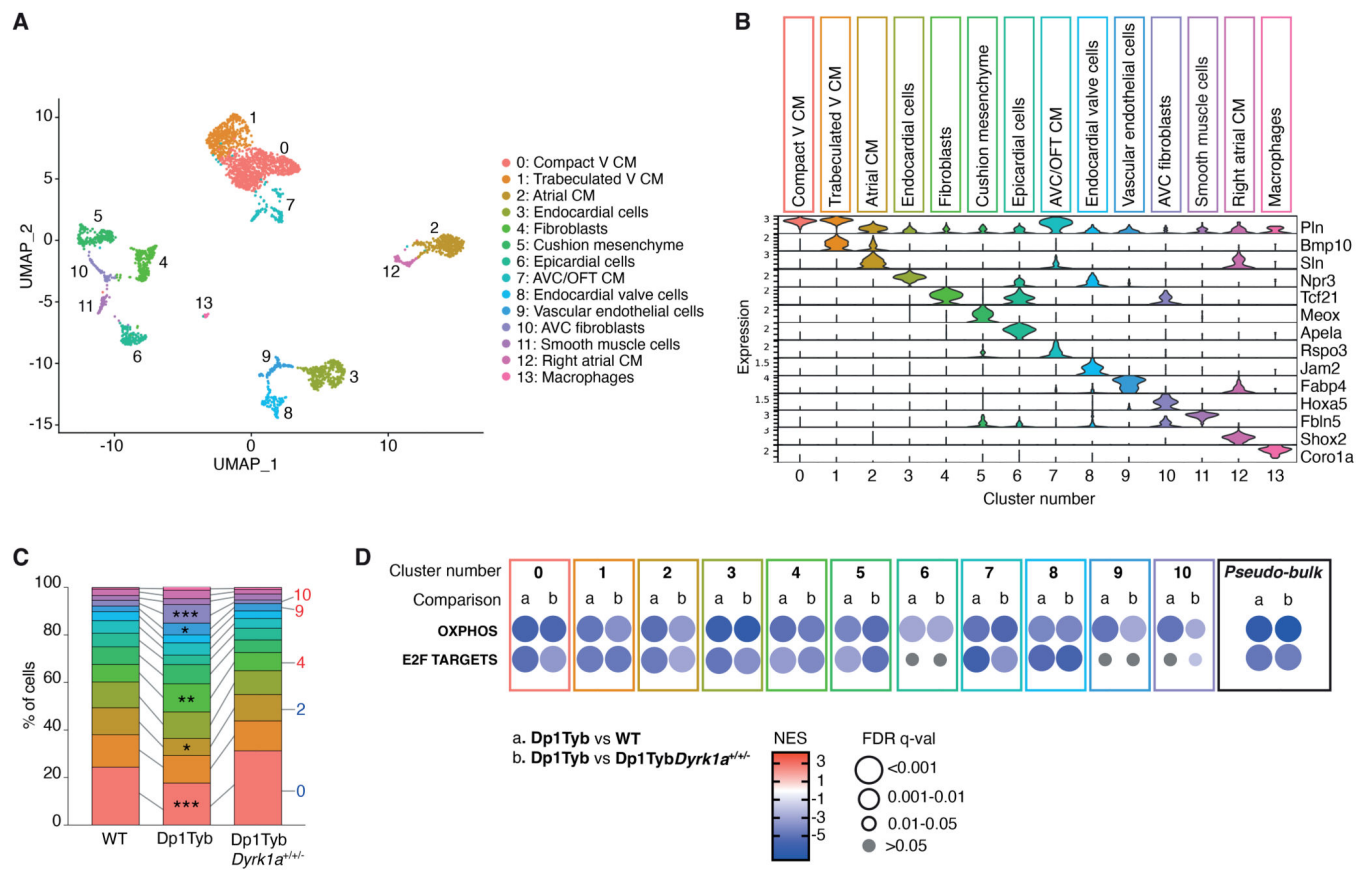
scRNAseq reveals similar gene expression changes across different cell types in Dp1Tyb mouse embryonic hearts. (**A**) Uniform Manifold Approximation and Projection (UMAP) clustering of scRNAseq data pooled from WT, Dp1Tyb and Dp1Tyb*Dyrk1a*^+/+/-^ E13.5 mouse hearts. Each dot represents a single cell; colors indicate 14 clusters whose identity was inferred based on expression of markers genes. V, ventricular; CM, cardiomyocytes; AVC, atrioventricular canal; OFT, outflow tract. (**B**) Violin plots showing the expression of representative marker genes across the 14 clusters; Y-axis shows the log-scale normalized read count. (**C**) Stacked column plot showing the percentage of cells in each of the 14 cell populations, colored according to cluster designation. Clusters with significantly altered percentages in Dp1Tyb hearts compared to WT are indicated; Fisher's exact test, * 0.01 < *P* < 0.05, ** 0.001 < *P* < 0.01, *** *P* < 0.001. **(D)** GSEA of Dp1Tyb versus WT (a) or Dp1Tyb versus Dp1Tyb*Dyrk1a*^+/+/-^ (b) scRNAseq data from the 11 most abundant clusters analyzed individually and pooled (pseudo-bulk), showing key pathways and their NES. Colors and sizes of circles indicate NES and FDR q-value, respectively. Sample numbers: *n*=2 WT, 1 Dp1Tyb, 2 Dp1Tyb*Dyrk1a*^+/+/-^ embryonic hearts.

Although RNAseq analysis identified dysregulated pathways, it was not able to specify which cell type was perturbed in Dp1Tyb hearts. To address this, we performed single cell RNAseq (scRNAseq) of WT and Dp1Tyb E13.5 mouse hearts and identified 14 clusters which were assigned to individual cell types based on expression of marker genes (Fig. 2A, B) (11). Many of the expected cell types were detected, including several sub-types of cardiomyocytes, endocardial cells, epicardial cells, fibroblasts, vascular endothelial cells, smooth muscle cells and macrophages (11). Comparison of the frequencies of each cell type showed that whereas all cell types were detected in each genotype (fig. S2), Dp1Tyb hearts had significantly reduced frequencies of ventricular and atrial cardiomyocytes and significantly increased frequencies of fibroblasts and vascular endothelial cells compared to WT hearts (*P*<0.05, Fig. 2C).

To determine which cell types showed the pathway changes seen in whole hearts, we subjected the scRNAseq data to GSEA. Initially, we pooled the scRNAseq across all cell types and found that this pseudo-bulk RNAseq analysis recapitulated the changes seen in the bulk RNAseq analysis, with Dp1Tyb mouse hearts showing decreased expression of oxidative phosphorylation and proliferation genes (Fig. 2D). Extending this analysis to the 11 most abundant cell clusters, we found that significantly decreased expression of the oxidative phosphorylation genes was seen in all cell types and significantly decreased expression of proliferation genes was seen in all cell types except epicardial cells, vascular endothelial cells, and atrioventricular cushion fibroblasts (*q*<0.05, Fig. 2D). Thus, decreased expression of oxidative phosphorylation and proliferation genes was seen across most cell types in Dp1Tyb mouse embryonic hearts.

### Dp1Tyb mouse embryonic hearts show proliferative defects

**Figure 3:**
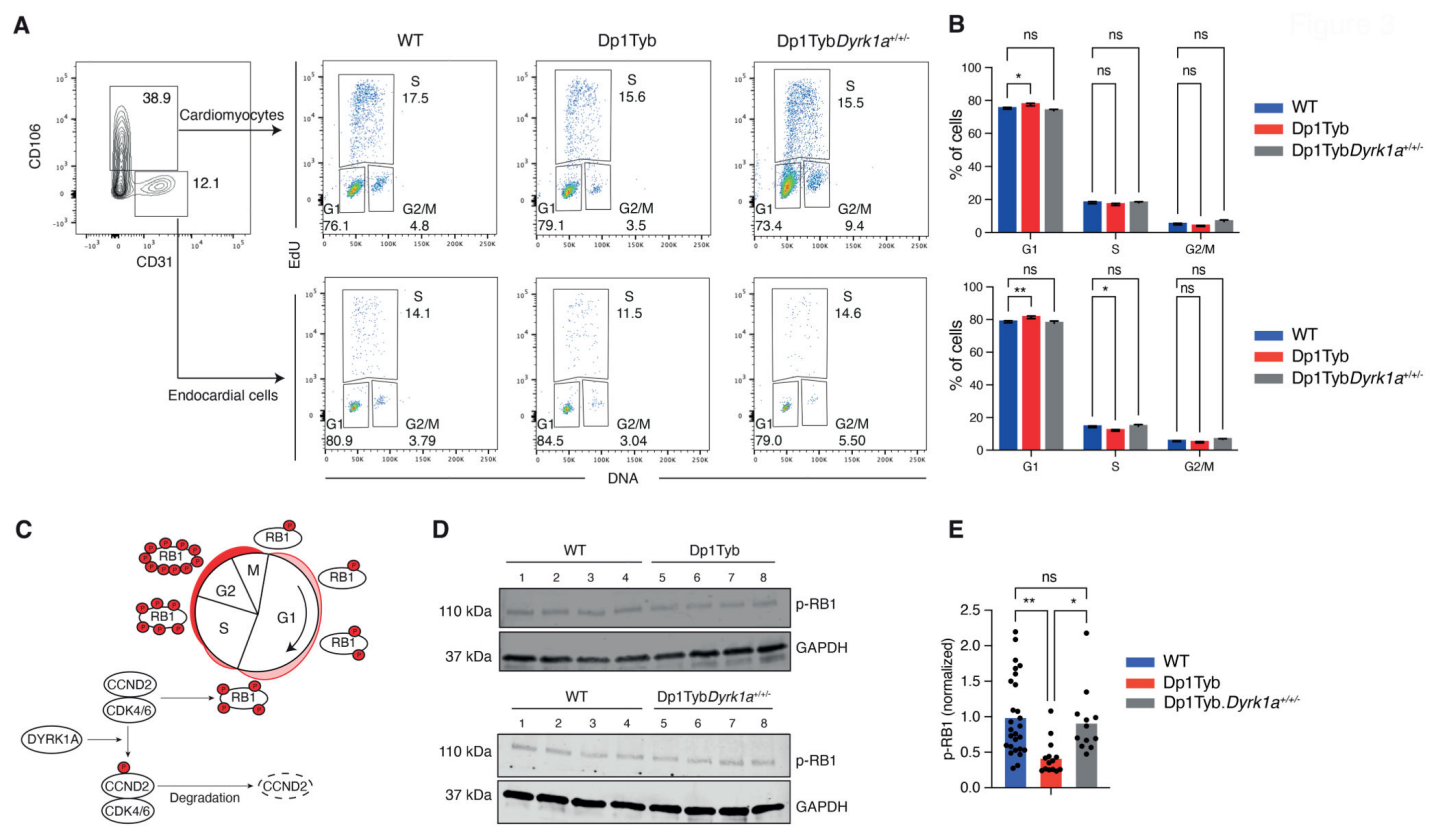
Proliferative defects in Dp1Tyb embryonic hearts. (**A**) Flow cytometric analysis of freshly isolated embryonic mouse hearts pulsed with EdU. EdU and DNA content of cardiomyocytes (CD106^+^CD31^-^) and endocardial cells (CD106^-^ CD31^+^) were used to distinguish cells in G1, S and G2/M phases. Numbers indicate percentage of cells in gates. (**B**) Mean (±SEM) percentage of cells in each cell cycle phase taken from data as in A. *n*=38 WT, 14 Dp1Tyb, 12 Dp1TybDyrk1a^+/+/-^ embryonic mouse hearts. (**C**) Diagram showing how DYRK1A may regulate the cell cycle. Cyclin D2 (CCND2) in complex with CDK4/6 phosphorylates RB1 promoting cell cycle progression. DYRK1A phosphorylates CCND2 leading to its degradation thereby causing reduced CDK4/6 activity, reduced RB1 phosphorylation and impaired cell cycle progression. (**D**) Representative immunoblot analysis of lysates from WT, Dp1Tyb and Dp1Tyb*Dyrk1a*^*+/+/-*^ E13.5 embryonic hearts probed with antibodies to phospho-RB1 (p-RB1) and GAPDH. Each lane represents an individual embryonic heart. (**E**) Mean p-RB1 abundance determined by immunoblots such as those in D, normalized to GAPDH and to the mean of WT samples which was set to 1. Dots represent individual embryos. *n*=27 WT, 14 Dp1Tyb, 12 Dp1TybDyrk1a^+/+/-^ embryonic hearts. Statistical significance was calculated using a Kruskal-Wallis test; * 0.01 < *P* < 0.05, ** 0.001 < *P* < 0.01; ns, not significant.

Although the decreased cell proliferation signature was present in both human DS fetal hearts and mouse Dp1Tyb hearts, this change was insufficient to cause heart defect pathology since it was present in Ts1Rhr hearts which do not show CHD. To investigate directly if cell proliferation was affected in Dp1Tyb mouse embryonic hearts, we measured the fraction of cells in different cell cycle phases. Flow cytometric analysis of Dp1Tyb mouse hearts showed an increased proportion of cardiomyocytes and endocardial cells in G1, and fewer cells in S phase, consistent with impaired proliferation (Fig. 3A-B). This proliferation defect may explain the decreased fraction of cardiomyocytes in Dp1Tyb hearts.

Pathway analysis of human DS and Dp1Tyb mouse hearts showed downregulated E2F target genes in both species (Fig. 1E, G). The E2F transcription factors control the expression of genes required for the G1 to S phase transition of the cell cycle (12). Inspection of the genes in the ‘leading edge’ of the E2F target gene set in the GSEA showed that there were 72 and 153 downregulated genes in human DS fetal hearts and Dp1Tyb embryonic hearts respectively, with 49 genes downregulated in common between the two species, indicating that downregulation of E2F activity may be the cause of the impaired proliferation (table S6). The E2F factors are repressed by binding to the Retinoblastoma protein RB1 (12). Phosphorylation of RB1 by CDK4 and CDK6 kinases in complex with Cyclin D proteins leads to dissociation of RB1 from E2F proteins allowing the latter to activate genes required for the G1 to S phase transition (Fig. 3C). To examine if the reduced E2F activity in Dp1Tyb hearts was due to impaired phosphorylation of RB1, we used immunoblotting to quantify amounts of phosphorylated RB1 (p-RB1). We found that Dp1Tyb mouse hearts had significantly decreased p-RB1 (*P*<0.05), which may account for reduced E2F activity and hence impaired cell proliferation (Fig. 3D, E).

### Dp1Tyb mouse embryonic cardiomyocytes have mitochondrial defects

**Figure 4:**
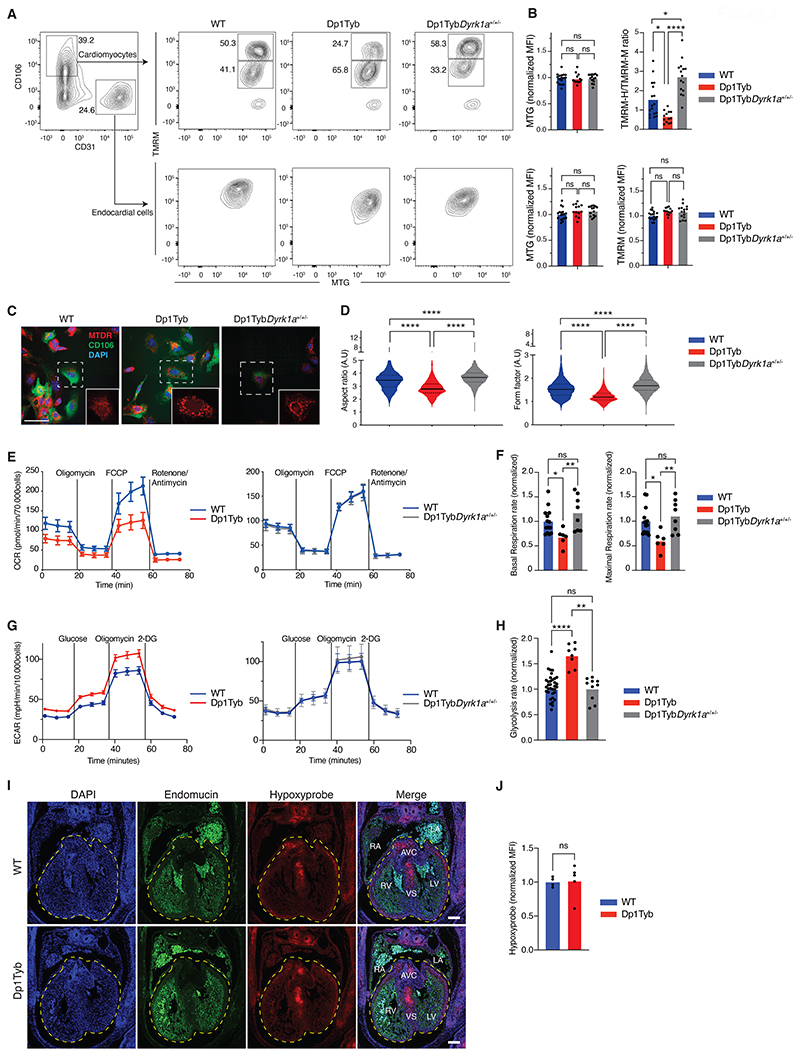
Mitochondrial defects in Dp1Tyb mouse embryonic cardiomyocytes. (**A**) Flow cytometric analysis showing gating strategy used to measure mitochondrial mass (MTG) and mitochondrial potential (TMRM) in cardiomyocytes (CD106^+^CD31^-^) and endocardial cells (CD106^-^CD31^+^) from E13.5 mouse embryonic hearts of the indicated genotypes. Cardiomyocytes were subdivided into cells that have a high (TMRM-H) and medium (TMRM-M) potential. Numbers indicate percentage of cells in gates. (**B**) Mean fluorescence intensity (MFI) of MTG and TMRM in endocardial cells and of MTG in cardiomyocytes normalized to the average of WT samples which was set to 1. For cardiomyocytes mitochondrial potential was measured using a TMRM-H/TMRM-M ratio. Dots represent individual embryos. *n*=17 WT, 13 Dp1Tyb, 15 Dp1TybDyrk1a^+/+/-^ embryonic hearts. (**C**) Representative confocal microscopy images of cells from E13.5 mouse hearts of the indicated genotypes showing staining with MitoTracker Deep Red (MTDR – mitochondria, red), anti-CD106 (cardiomyocytes, green) and DAPI (blue). Images show a maximum projection of Z-stacks from 0 to 3 μm with a step size of 1 μm. Insets are enlarged images of the region in the dashed square showing the mitochondrial network. Scale bar 50 μm. (**D**) Violin plots of mitochondrial aspect ratio and form factor in cardiomyocytes (CD106^+^) determined from images such as those in C (fig. S3A, B). Aspect ratio and form factor are measures of distortion from circularity and degree of branching, respectively (54). Black lines indicate median, dotted lines indicate 25th and 75th centiles. *n*=25 WT, 10 Dp1Tyb, 17 Dp1TybDyrk1a^+/+/-^ mouse embryonic hearts. (**E**) Mean±SEM oxygen consumption rate (OCR) in E13.5 mouse heart cells from embryos of the indicated genotypes analyzed using a Seahorse analyzer with oligomycin (ATP synthase inhibitor), FCCP (depolarizes mitochondrial membrane potential), and rotenone and antimycin (complex I and III inhibitors) added at the indicated times. Basal respiration rate was calculated from the mean of the first three measurements, maximal respiration rate from the three time points after addition of FCCP. (**F**) Mean basal and maximal respiration rates of E13.5 mouse heart cells normalized to the mean rates in WT hearts. Dots represent individual embryos. *n*=14 WT, 6 Dp1Tyb, 8 Dp1Tyb*Dyrk1a*^+/+/-^ embryonic hearts. (**G**) Mean±SEM extracellular acidification rate (ECAR) in E13.5 mouse heart cells from embryos of the indicated genotypes analyzed using a Seahorse analyzer with glucose, oligomycin (ATP synthase inhibitor) and 2 deoxy-glucose (2-DG, competitive inhibitor of glucose) added at the indicated times. Glycolysis rate was calculated as the difference between the mean ECAR of the three measurements before and after glucose injection. (**H**) Mean glycolysis rates of E13.5 mouse heart cells normalized to the mean rates in WT hearts. Dots represent individual embryos. *n*=35 WT, 8 Dp1Tyb, 10 Dp1TybDyrk1a^+/+/-^ embryonic hearts. (**I**) Representative images of sections of E13.5 mouse hearts of the indicated genotypes showing a 4-chamber view stained with anti-Endomucin (endothelial cells, green), anti-Hypoxyprobe (hypoxia, red) and DAPI (blue). Dashed line indicates a region of interest (ROI) encompassing the ventricles and the atrioventricular cushions. Scale bar 200 μm. (**J**) MFI of anti-Hypoxyprobe in ROI determined from images such as those in I. *n*=4 WT, 6 Dp1Tyb mouse embryonic hearts. Dots represent individual embryos. LV, left ventricle; RV, right ventricle; LA, left atrium; RA, right atrium; AVC, atrioventricular cushion; VS, ventricular septum. Statistical significance was calculated using a Kruskal-Wallis (B, D, F, H) or Mann Whitney test (J), * 0.01 < *P* < 0.05, ** 0.001 < *P* < 0.01, **** *P* < 0.0001; ns, not significant.

To determine whether the decreased expression of oxidative phosphorylation genes indicated impaired mitochondrial respiration, we used flow cytometry to measure mitochondrial mass and the mitochondrial inner membrane potential of Dp1Tyb mouse hearts. Mitochondrial mass was not altered in either cardiomyocytes or endocardial cells, and mitochondrial potential was unchanged in endocardial cells, however, the ratio of cells with high to intermediate membrane potential was significantly reduced (*P*<0.05) in cardiomyocytes from E13.5 Dp1Tyb mouse hearts (Fig. 4A, B). Decreased mitochondrial membrane potential suggested that the maturation of Dp1Tyb mouse cardiomyocytes may be affected. A developmental transition, typically occurring at E11.5 in the mouse, is characterized by closure of the mitochondrial permeability transition pore, thereby elevating mitochondrial potential, and by elongation and branching of mitochondria (13). Confocal microscopy imaging showed that mitochondria in cardiomyocytes from Dp1Tyb mouse E13.5 hearts had significantly lower aspect ratios and form factors (*P*<0.05) compared to those in WT mouse cardiomyocytes, indicating that Dp1Tyb mitochondria are less elongated and branched, consistent with impaired cardiomyocyte maturation (Fig. 4C-D, fig. S3A-B). To directly analyze mitochondrial function, we measured mitochondrial respiration of Dp1Tyb mouse embryonic hearts. We found that cells from E13.5 Dp1Tyb mouse hearts had significantly reduced basal and maximal respiration rates (*P*<0.05), consistent with impaired mitochondrial function (Fig. 4E-F). Furthermore, analysis of glycolysis rates showed that cells from Dp1Tyb hearts had significantly increased rates of glycolysis (*P*<0.05), demonstrating a bioenergetic switch away from oxidative phosphorylation (Fig. 4G-H).

One possible cause for this switch towards glycolysis could be hypoxia, which through HIF-1α results in the upregulation of genes encoding enzymes in the glycolytic pathway (14). To investigate if increased hypoxia could be playing a role in this altered cardiac metabolism, we measured hypoxia in developing Dp1Tyb and control embryonic hearts by injecting pregnant mice with pimonidazole hydrochloride, also known as Hypoxyprobe (15). Pimonidazole covalently binds to thiol groups on proteins and amino acids in hypoxic cells and can be visualized with an antibody. This analysis showed no change in the amount of hypoxia in Dp1Tyb mouse hearts compared to WT mouse hearts (Fig. 4I-J). This result was further supported by GSEA which did not show any significant changes (*P*>0.05) in the expression of HIF-1α-regulated genes (table S6). Thus, increased hypoxia is unlikely to be the cause of the increased glycolysis. Taken together these results demonstrate that Dp1Tyb mouse embryonic hearts have impaired mitochondrial respiration, with a compensatory increase in glycolysis.

### Three copies of *Dyrk1a* are necessary to cause heart defects

**Figure 5:**
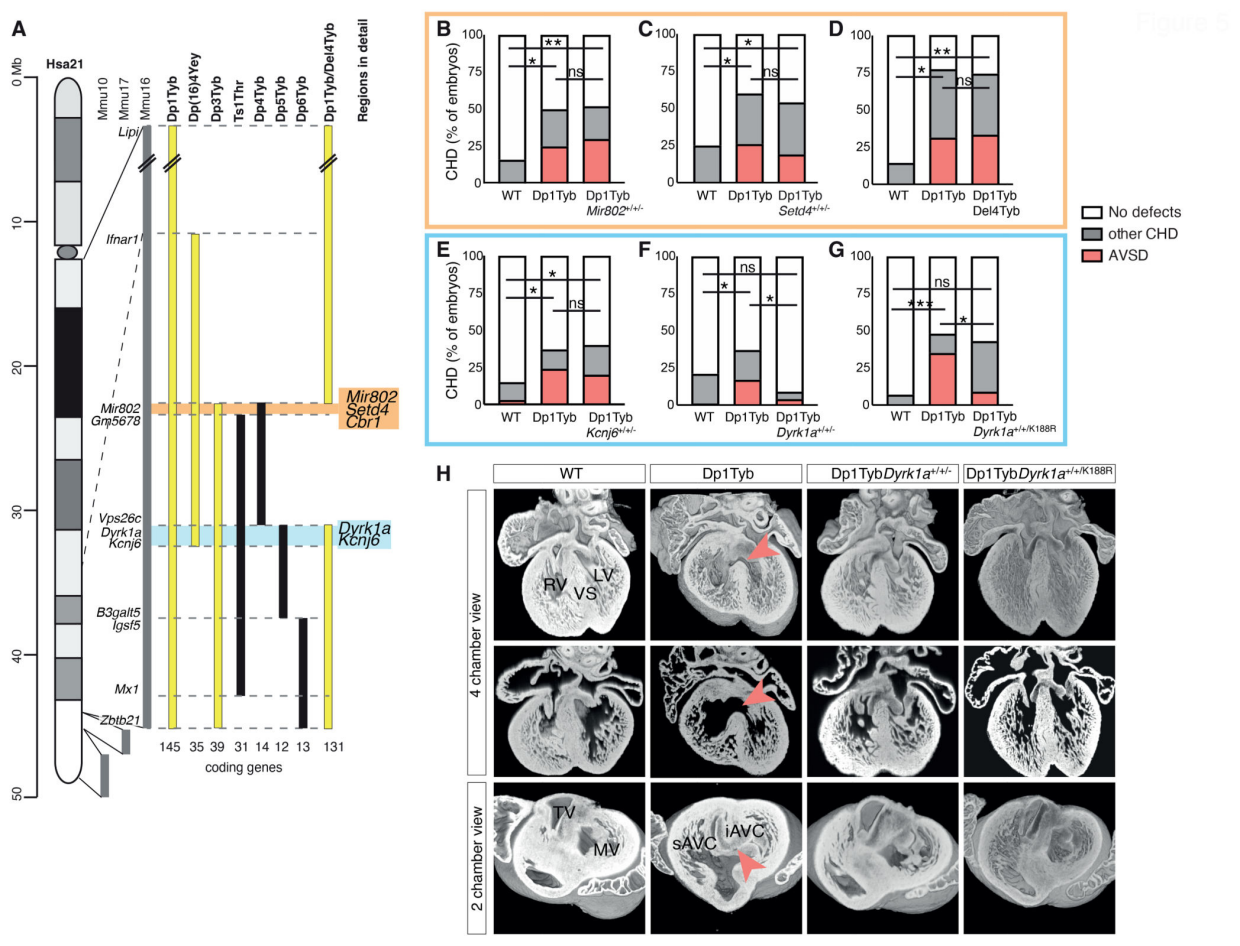
Three copies of *Dyrk1a* are necessary to cause heart defects. (**A**) Map of Hsa21 showing regions of orthology to Mmu10, Mmu17 and Mmu16 (grey) and indicating regions of Mmu16 that are duplicated in mouse strains that show CHD (yellow) and in those that do not (black); genes at boundaries of these duplications are indicated next to the Mmu16 map; numbers of coding genes indicated below duplicated regions. Two genetic intervals containing 3 and 2 candidate genes for CHD are indicated in orange and blue, respectively. (**B-G**) Graphs of percentage of CHD in E14.5 mouse embryonic hearts from the indicated mouse models, indicating the frequency of AVSD and other CHD, which are predominantly VSDs and occasionally outflow tract defects such as overriding aorta. The orange and blue boxes highlight the genes found in the 2 candidate regions. The cross of Dp1Tyb to Del4Tyb was used to determine if 3 copies of *Cbr1* are required for heart defects. Numbers of embryonic hearts analyzed: (B) WT (*n*=25), Dp1Tyb (*n*=20), Dp1Tyb*Mir802*^+/+/-^ (*n*=27) hearts with two copies of *Mir802*; (C) WT (*n*=24), Dp1Tyb (*n*=23), Dp1Tyb*Setd4*^+/+/-^ (*n*=26) hearts with two copies of *Setd4*; (D) WT (*n*=7), Dp1Tyb (*n*=13), Dp1TybDel4Tyb (*n*=15) hearts with two copies of the region deleted in Del4Tyb; (E) WT (*n*=34), Dp1Tyb (*n*=41), Dp1Tyb*Kcnj6*^+/+/-^ (*n*=30) hearts with two copies of *Kcnj6*; (F) WT (*n*=91), Dp1Tyb (*n*=86), Dp1Tyb*Dyrk1a*^+/+/-^ (*n*=23) hearts with two copies of *Dyrk1a*; (G) WT (*n*=30), Dp1Tyb (*n*=23), Dp1Tyb*Dyrk1a*^+/+/K188R^ (*n*=44) hearts with two WT and one kinase-inactive allele of *Dyrk1a*. Fisher's exact test, * 0.01 < *P* < 0.05, ** 0.001 < *P* < 0.01, *** *P* < 0.001 for difference in number of total CHD, except for Dp1Tyb*Dyrk1a*^+/+/K188R^ cohort, where statistics were calculated for number of AVSD; ns, not significant. (**H**) 3 Dimensional high resolution episcopic microscopy (HREM) rendering of WT, Dp1Tyb, Dp1Tyb*Dyrk1a*^+/+/-^ and Dp1Tyb*Dyrk1a*^+/+/K188R^ mouse hearts, eroded to show an anterior four-chamber view (top and middle) and a two-chamber view (bottom) seen from the atria at the level of the atrioventricular canal. Top and bottom rows show eroded 3D views, middle row shows 2D sections at the same level as shown in the top row. Red arrowheads indicate VSD (top, middle) or AVSD (bottom). iAVC, inferior atrio-ventricular cushion; LV, left ventricle; MV, mitral valve; RV, right ventricle; sAVC, superior atrio-ventricular cushion; TV, tricuspid valve; VS, ventricular septum.

To gain further insight into the basis of CHD in DS we used systematic genetic mapping to identify a causative gene responsible for this pathology. Using a panel of mouse strains with a nested series of duplications of regions on Mmu16, we previously showed that a region of 39 genes duplicated in Dp3Tyb mice was sufficient to cause CHD (8). However, when this was broken down into three shorter duplicated regions in Dp4Tyb, Dp5Tyb and Dp6Tyb mice, none of the resulting strains had heart defects, implying that there must be at least two causative genes (Fig. 5A) (8). Furthermore, since Ts1Rhr mouse embryos do not have CHD, one of the causative genes must be within the 8 coding genes and 1 microRNA gene that are duplicated in Dp3Tyb but not Ts1Rhr mice (Fig. 5A). Another study showed that Dp(16)4Yey mice with an extra copy of a 35-gene region on Mmu16 which partially overlaps Dp3Tyb, also have CHD (16). This overlap includes 16 genes at the proximal end of the Dp3Tyb duplication covering the entire Dp4Tyb region and the first two genes within the Dp5Tyb region. Taken together, the simplest explanation for these results is that one causative gene lies within the proximal region duplicated in Dp3Tyb mice but not Ts1Rhr mice, containing two coding genes (*Setd4, Cbr1*) and a microRNA gene (*Mir802*) (Fig. 5A, orange), and a second causative gene lies within the first two genes of the Dp5Tyb region (*Dyrk1a, Kcnj6*) (Fig. 5A, blue). Thus, we tested the potential role for each of these 5 genes in causing CHD, by reducing its copy number in Dp1Tyb embryos from three to two.

We crossed Dp1Tyb mice to mouse strains deficient in each of the candidate genes, except for *Cbr1*, which we tested by crossing to Del4Tyb mice that have a deletion of the entire Dp4Tyb region (Fig. 5A, fig. S4A). For each cross we analyzed the hearts of E14.5 embryos using high resolution episcopic microscopy (HREM) (17) to generate detailed 3D images to accurately identify different types of CHD. As expected, compared to WT controls, Dp1Tyb embryos had increased rates of CHD, especially the more severe AVSD, which are typified by a common atrioventricular valve in place of separated mitral and tricuspid valves (Fig. 5B-H). Removing one copy of *Mir802, Setd4, Cbr1* or *Kcnj6* did not affect the frequency of CHD in general, or specifically AVSDs (Fig. 5B-E). However, reducing the copy number of *Dyrk1a* from three to two completely rescued CHD in Dp1Tyb*Dyrk1a*^+/+/-^ mice (Fig. 5F, H). Thus, three copies of *Dyrk1a* are necessary to cause CHD in Dp1Tyb mice.

*Dyrk1a* encodes the DYRK1A serine/threonine protein kinase. To investigate whether increased DYRK1A kinase activity is required for CHD, we crossed Dp1Tyb mice to a strain carrying an allele coding for kinase-inactive DYRK1A (*Dyrk1a*^K188R^). The resulting Dp1Tyb*Dyrk1a*^+/+/K188R^ embryos had no increase in AVSDs compared to WT controls, and significantly fewer (*P*=0.0141) AVSDs than Dp1Tyb embryos (Fig. 5G-H). Thus, increased DYRK1A catalytic kinase activity is necessary for severe CHD in Dp1Tyb mice.

### Increased dosage of *Dyrk1a* causes key transcriptional changes in Dp1Tyb embryonic hearts

**Figure 6:**
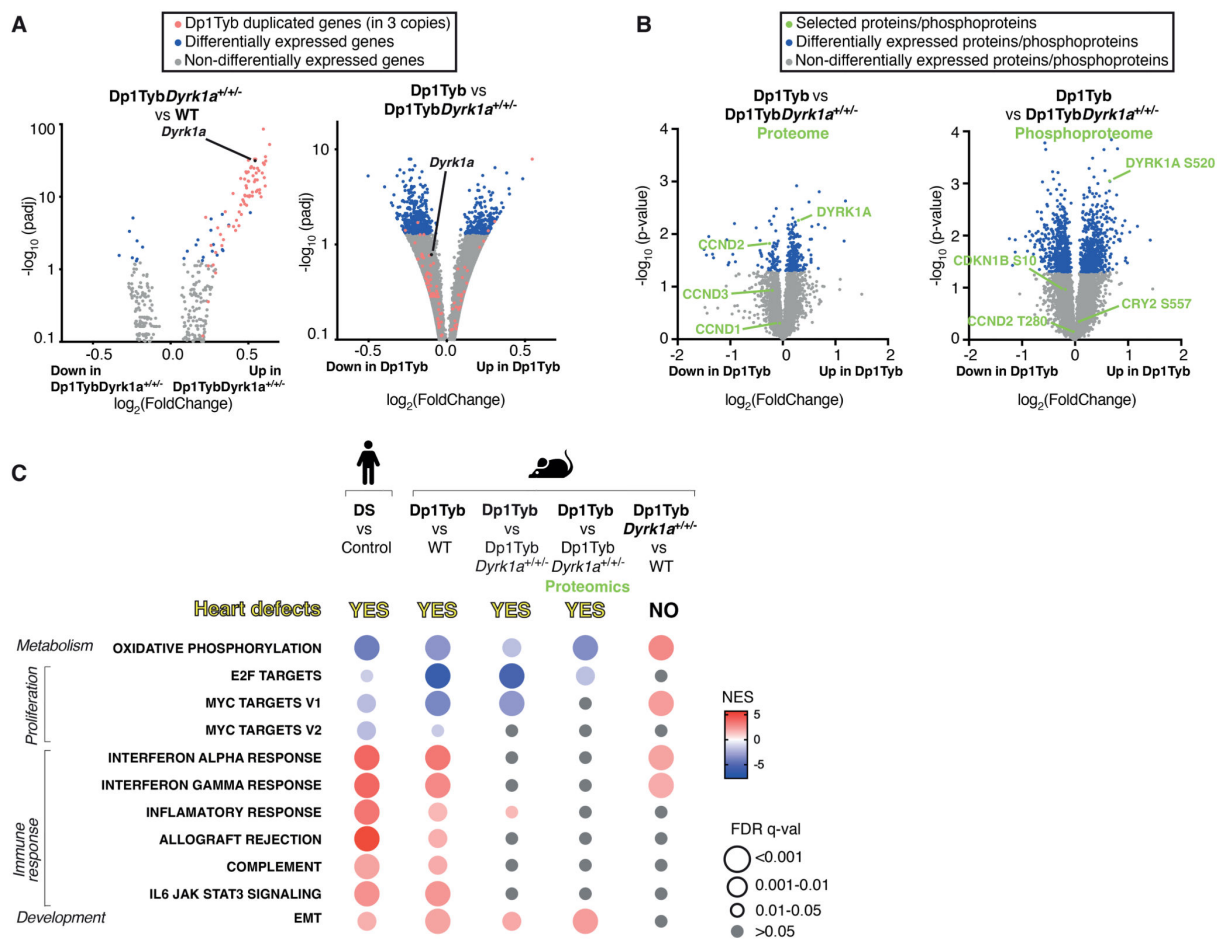
Increased dosage of *Dyrk1a* causes key transcriptional changes in Dp1Tyb mouse embryonic hearts. (**A**) Volcano plots showing fold-change in gene expression in E13.5 mouse embryonic hearts, Dp1Tyb*Dyrk1a*^+/+/-^ versus WT (left) and Dp1Tyb versus Dp1Tyb*Dyrk1a*^+/+/-^ (right), plotted against adjusted *P*-value for significance of the difference. Genes present in three copies in Dp1Tyb mice (red) and differentially expressed genes (blue) and *Dyrk1a* (black) are indicated. (**B**) Volcano plots showing fold-change in abundance of proteins and phosphorylated sites in Dp1Tyb versus Dp1Tyb*Dyrk1a*^+/+/-^ E13.5 hearts. DYRK1A, CCND1, CCND2 and CCND3 are indicated in green on the proteome plot (left); phosphorylated sites known to be DYRK1A targets and an autophosphorylation site on DYRK1A are indicated in green on the phosphoproteome plot (right). (**C**) Comparison of dysregulated pathways determined by GSEA of RNAseq and proteomic experiments. Colors and sizes of circles indicate NES and FDR q-value, respectively. Sample numbers: *n*=5 embryonic hearts.

Next, we investigated whether increased dosage of *Dyrk1a* was responsible for the transcriptional changes in Dp1Tyb mouse E13.5 embryonic hearts. A comparison of the transcriptomes of WT, Dp1Tyb and Dp1Tyb*Dyrk1a*^+/+/-^ hearts showed that compared to WT controls, Dp1Tyb*Dyrk1a*^+/+/-^ samples had increased expression of the duplicated genes, but very few other differentially expressed genes (Fig. 6A, table S7). In contrast, comparison of Dp1Tyb and Dp1Tyb*Dyrk1a*^+/+/-^ mouse hearts showed large numbers of differentially expressed genes, as had been seen in the comparison of Dp1Tyb with WT controls (Fig. 6A). These results show that an extra copy of *Dyrk1a* was responsible for most of the transcriptional changes in developing Dp1Tyb mouse hearts. We extended this analysis to the proteome using mass spectrometric analysis of Dp1Tyb and Dp1Tyb*Dyrk1a*^+/+/-^ mouse E13.5 hearts, revealing 290 differentially expressed proteins (Fig. 6B, table S8). As expected, the amount of DYRK1A was about 1.5-fold higher in Dp1Tyb compared to Dp1Tyb*Dyrk1a*^+/+/-^ hearts. Moreover, pathway analysis of the transcriptomic and proteomic data showed that 3 copies of *Dyrk1a* are necessary for the decreased expression of oxidative phosphorylation and proliferation genes and increased expression of EMT genes, but not for the increased expression of immune response genes (Fig. 6C, fig. S1D, E). A comparison of the differentially expressed genes in Dp1Tyb versus WT mouse hearts and Dp1Tyb versus Dp1Tyb*Dyrk1a*^+/+/-^ hearts, showed that there were 32 upregulated and 46 downregulated genes in common between these comparisons, representing genes most likely to be regulated by increased *Dyrk1a* dosage (table S9). STRING protein interaction (https://string-db.org/) analysis of these genes once again showed a clear signature of decreased networks of proteins involved in proliferation (table S9).

Furthermore, scRNAseq analysis of Dp1Tyb*Dyrk1a*^+/+/-^ mouse E13.5 hearts showed that reduction of the copy number of *Dyrk1a* reversed the decreased fraction of cardiomyocytes and the increased fraction of fibroblasts seen in Dp1Tyb hearts (Fig. 2C, fig. S2), demonstrating that these changes in cell type abundance are dependent on three copies of *Dyrk1a*. Moreover, GSEA pathway analysis showed that changes in expression of oxidative phosphorylation, proliferation and EMT genes which were seen across most cell types, were also dependent on 3 copies *Dyrk1a* (Fig. 2D).

### *Dyrk1a* is broadly expressed in many cardiac cell types

**Figure 7:**
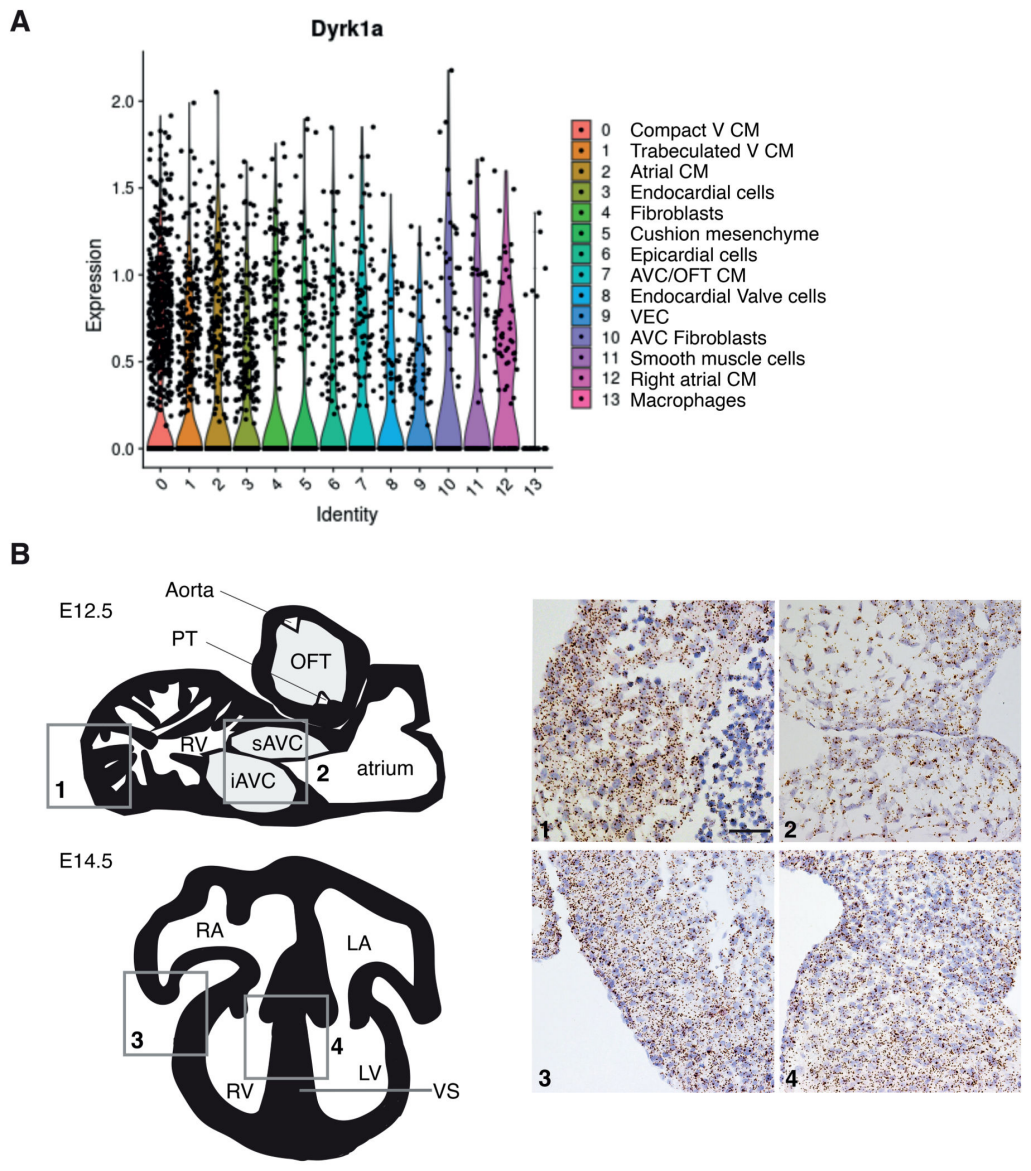
*Dyrk1a* expression in a broad range of cell types in the developing mouse heart. **(A)** Violin plots showing expression of *Dyrk1a* in mouse E13.5 hearts across 14 cell clusters identified in Fig. 2B. Dots indicate single cells. Sample numbers: *n*=5 embryonic hearts. (**B**) Left, schematic illustrations of a sagittal section (top) of an E12.5 mouse heart and a 4-chamber view (bottom) of an E14.5 heart. Right, RNAscope analysis of *Dyrk1a* expression (brown dots) in sections of the right ventricle myocardial wall (1) and atrioventricular cushions (2) at E12.5 and right ventricular myocardial wall (3) and ventricular septum (4) at E14.5; sections were counterstained with hematoxylin (blue). Scale bar 50 μm. iAVC, inferior atrioventricular cushion; LA, left atrium; LV, left ventricle; OFT, outflow tract; PT, pulmonary trunk; RA, right atrium; RV, right ventricle; sAVC, superior atrioventricular cushion; VS, ventricular septum.

Since a third copy of *Dyrk1a* had a profound effect on the transcriptomes of most cell types in the developing heart, we examined its expression pattern. Using the scRNAseq data, we found that *Dyrk1a* was expressed in all cell types in E13.5 hearts, a conclusion that was further supported by RNAscope analysis of E12.5 and E14.5 hearts, an in situ hybridization method that detects mRNA in tissue sections (Fig. 7A-B). This broad expression of *Dyrk1a* is consistent with a role for increased DYRK1A causing the observed pathway changes across multiple cell types.

### Increased dosage of *Dyrk1a* is necessary for reduced proliferation in Dp1Tyb mouse embryonic hearts

Next, we investigated whether increased dosage of *Dyrk1a* was also required for the physiological changes seen in Dp1Tyb mouse embryonic hearts. Analysis of proliferation showed that Dp1Tyb*Dyrk1a*^+/+/-^ mouse cardiomyocytes and endocardial cells had no change in the fraction of cells in G1, S and G2/M phases compared to WT cells, demonstrating that 3 copies of *Dyrk1a* were necessary for the impaired proliferation (Fig. 3A, B). Furthermore, the reduction in phosphorylated RB1 in Dp1Tyb mouse embryonic hearts was also reversed by reducing the copy number of *Dyrk1a* from 3 to 2 (Fig. 3D-E), and Dp1Tyb*Dyrk1a*^+/+/-^ hearts no longer showed reduced expression of E2F target genes (Fig. 6C, table S6).

Increased expression of DYRK1A may cause decreased cell proliferation by phosphorylating Cyclin D (CCND) proteins, leading to their degradation (Fig. 3C) (18–22). To investigate whether this was occurring in Dp1Tyb mouse hearts we used mass spectrometry to compare the phospho-proteomes of Dp1Tyb and Dp1Tyb*Dyrk1a*^+/+/-^ E13.5 hearts, showing that there were many phospho-peptides whose abundance differed significantly (*P*<0.05) because of a third copy of *Dyrk1a* (Fig. 6B, table S8). This included increased pS520-DYRK1A, an autophosphorylation site of the kinase, consistent with increased DYRK1A activity in Dp1Tyb compared to Dp1Tyb*Dyrk1a*^+/+/-^ hearts (23). However, we saw no change in the abundance of pT280-CCND2, a site reported to be phosphorylated by DYRK1A in cardiomyocytes (Fig. 6B) (20). Furthermore, we were able to detect only two other known DYRK1A phosphorylation targets, pS10-CDKN1B and pS557-CRY2, neither of which was altered in abundance. However, analysis of the whole proteome showed a decreased abundance of CCND2 in Dp1Tyb hearts (*P*<0.05), and decreased abundance of CCND1 and CCND3, though these latter changes were not significant (*P*>0.05) (Fig. 6B). These results are consistent with increased dosage of DYRK1A impairing proliferation in Dp1Tyb embryonic hearts through phosphorylation and subsequent degradation of CCND2, leading to reduced CDK4/6-induced phosphorylation of RB1 and hence less expression of E2F-regulated genes that are required for G1 to S phase transition.

### Three copies of *Dyrk1a* cause mitochondrial dysfunction in embryonic cardiomyocytes

Next, we examined whether the mitochondrial dysfunction in Dp1Tyb mouse cardiomyocytes was dependent on 3 copies of *Dyrk1a*. We found that reduction of the copy number of *Dyrk1a* from 3 to 2 reversed the decreased mitochondrial potential and impaired mitochondrial morphology in Dp1Tyb mouse cardiomyocytes (Fig. 4A-D). Furthermore, cells from Dp1Tyb*Dyrk1a*^+/+/-^ mouse E13.5 hearts showed normal rates of basal and maximal respiration and glycolysis (Fig. 4E-H).

### Pharmacological inhibition of DYRK1A results in a partial reversal of transcriptional changes in Dp1Tyb mouse embryos

Finally, we investigated whether treatment of pregnant mice with an inhibitor of DYRK1A kinase activity could reverse the CHD in Dp1Tyb mouse embryos. To investigate this we used Leucettinib-21, a recently developed DYRK1A inhibitor and a negative control, iso-Leucettinib-21, an inactive isomer (fig. S5A). Leucettinib-21 is a potent inhibitor of DYRK1A (IC_50_ = 3.1 nM), whereas iso-Leucettinib-21 has an IC_50_ >10 μM for DYRK1A (24). First, we asked if Leucettinib-21 administered to the pregnant mouse could pass through the placenta into the embryo. Analysis of mice treated with 0.3, 3 or 30 mg/kg of Leucettinib-21 showed detectable Leucettinib-21 in the embryo at 3 and 30 but not 0.3 mg/kg (Fig. S5B). Based on the measured amounts of Leucettinib-21 in the embryo, the 3 and 30 mg/kg doses resulted in around 24 nM and 560 nM Leucettinib-21 in the embryo. We chose to use 30 mg/kg Leucettinib-21 to maximize the chance of observing an effect.

**Figure 8:**
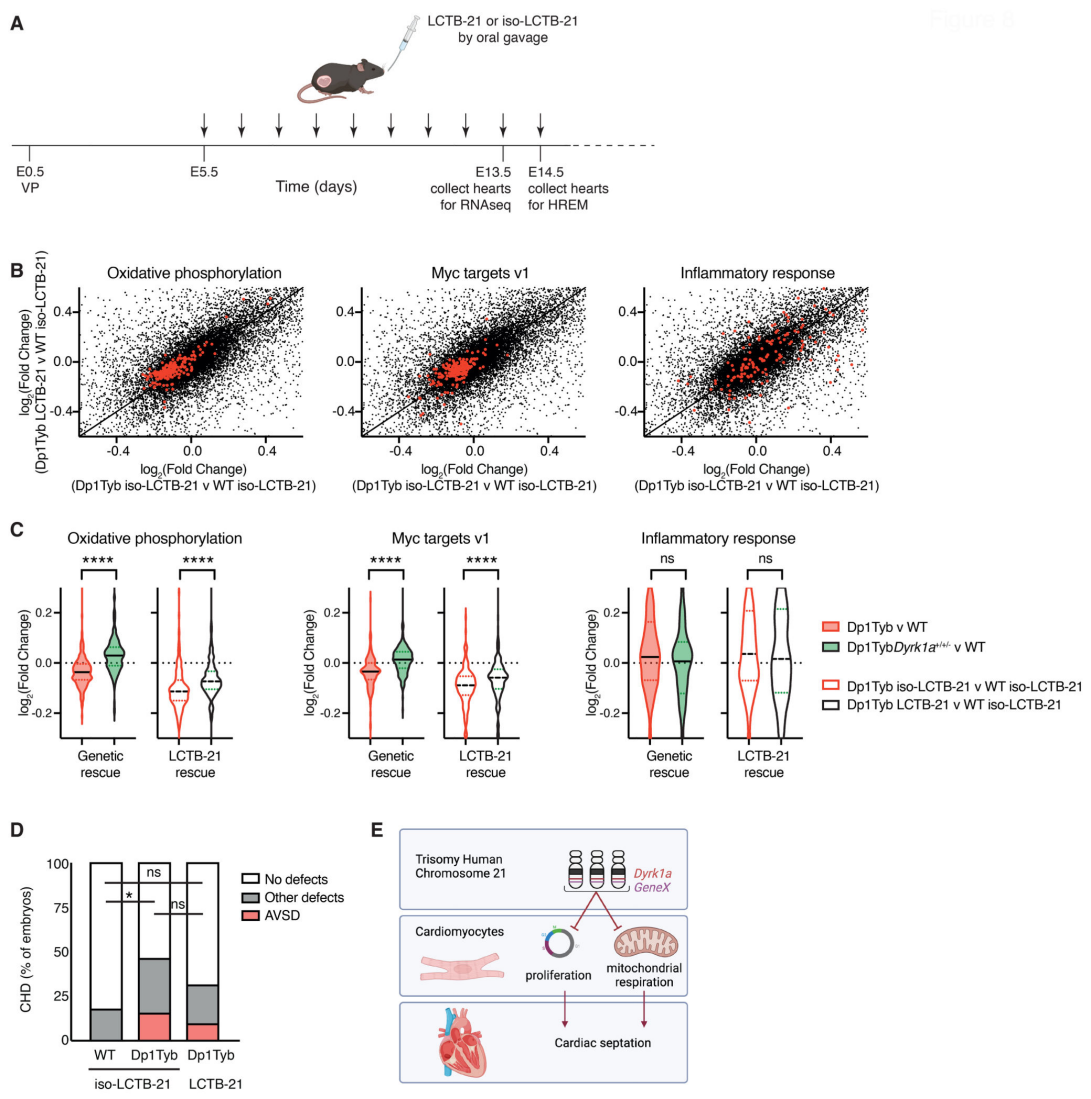
Pharmacological inhibition of Dyrk1a partially rescues CHD in the Dp1Tyb mouse model of DS. (**A**) C57BL/6J females that had been mated with Dp1Tyb males, were treated daily (vertical arrows) by oral gavage with Leucettinib-21 (LCTB-21) or iso-Leucettinib-21 (iso-LCTB-21) from 5 days after vaginal plug (VP) was found (embryonic day 0.5, E0.5). Embryos were collected at E13.5 for RNAseq, or at E14.5 for HREM. (**B**) Scatter plots comparing the log_2_(fold-change) (Log2FC) of mRNA expression for all genes (black) for Dp1Tyb versus WT embryos both treated with iso-LCTB-21 or Dp1Tyb embryos treated with LCTB-21 versus WT embryos treated with iso-LCTB-21. Genes from the Hallmark genesets for Oxidative Phosphorylation (left), Myc targets V1 (middle) and inflammatory response (right), are highlighted in red. *n*=5 for each condition. (**C**) Violin plots showing the Log2FC of expression of the same genesets as in B, Oxidative Phosphorylation (left), Myc targets V1 (middle), and inflammatory response (right), for the following comparisons: untreated Dp1Tyb versus WT, untreated Dp1TybDyrk1a^+/+/-^ versus WT, Dp1Tyb treated with iso-LCTB-21 versus WT treated with iso-LCTB-21 and Dp1Tyb treated with LCTB-21 versus WT treated with iso-LCTB-21. Black lines indicate median, dotted lines indicate 25th and 75th centiles. Dotted line at 0 indicates no change. *n*=5 for each condition. (**D**) Graph of percentage of CHD in E14.5 mouse embryonic hearts from the indicated models. Number of hearts analyzed: WT (*n*=34) and Dp1Tyb (*n*=26) treated with iso-LCTB-21, and Dp1Tyb treated with LCTB-21 (*n*=32). (**E**) Three copies of *Dyrk1a* and a second unknown gene (*GeneX*) lead to impaired proliferation and mitochondrial respiration in cardiomyocytes which is required for correct septation of the heart. Created in Biorender. Statistical tests were carried out with a Kruskal-Wallis (C) or Fisher's exact (D) test; * 0.01 < *P* < 0.05, **** *P* < 0.0001; ns, not significant.

We treated pregnant mice with Leucettinib-21 or iso-Leucettinib-21 daily starting at E5.5, which is before the heart forms, until E13.5 for RNAseq analysis and E14.5 for analysis of CHD by HREM (Fig. 8A). Homozygous genetic deletion of *Dyrk1a* results in a mid-gestation lethality, with no embryos surviving beyond E13.5 (25). Thus, it was possible that Leucettinib-21 treatment would cause embryonic lethality. However, we found that treatment of mated mice with 30 mg/kg Leucettinib-21 did not affect the fraction of mice that became pregnant or the size of their litters (fig. S5C-D). Furthermore, the recovery of Dp1Tyb embryos at E14.5 was also not affected (fig. S5E), in line with the normal segregation of the mutation, as previously reported (8). Thus, Leucettinib-21 treatment does not interfere with pregnancy and is likely only partially inhibiting DYRK1A.

RNAseq analysis of E13.5 embryos from mice treated with Leucettinib-21 or iso-Leucettinib-21 showed that the active Leucettinib-21 inhibitor partially reversed the decreased expression of genes in the oxidative phosphorylation and proliferation (Myc targets v1) pathways in Dp1Tyb embryonic hearts, however, it had no effect on the increased expression of inflammatory genes in the mutant hearts (Fig. 8B, table S10). The changes in expression caused by Leucettinib-21 were qualitatively similar to the changes seen when the copy number of *Dyrk1a* was reduced from 3 to 2 in Dp1Tyb embryos but were smaller in magnitude, consistent with Leucettinib-21 causing a partial inhibition of DYRK1A in the embryonic heart (Fig. 8C). Lastly, we used HREM to analyze the embryos for CHD. We found that Leucettinib-21 treatment resulted in a frequency of CHD in Dp1Tyb embryos that was between that seen in WT and Dp1Tyb embryos treated with iso-Leucettinib-21, but was not significantly different from either (*P*>0.05) (Fig. 8D). This is consistent with the possibility that inhibition of DYRK1A results in a partial rescue of the DYRK1A-dependent CHD in Dp1Tyb embryos but more work would be required to establish this.

## Discussion

Here we show that heart tissue from human fetuses with DS have characteristic transcriptional changes, many of which are shared with embryonic hearts from the Dp1Tyb and Dp3Tyb mouse models of DS that show CHD. Gene set enrichment analysis identified several pathways that were altered in the hearts of both human DS and mouse models. Of these, decreased expression of oxidative phosphorylation genes correlated most strongly with CHD in the mouse models, suggesting that impaired mitochondrial function may be an important cause of the developmental defects. In agreement with our results, decreased expression of oxidative phosphorylation genes in human DS fetal hearts has been previously seen using microarray technology (26). In addition, physiological analysis demonstrated reduced mitochondrial membrane potential and respiration in Dp1Tyb mouse embryonic cardiomyocytes. Decreased expression of proliferation genes partially correlated with CHD, being seen in human DS hearts, and mouse Dp1Tyb and Dp3Tyb hearts. Hearts from the Ts1Rhr mouse strain which does not have CHD, also showed decreased expression of proliferation genes, but only in two out of three gene sets compared to a decrease in all three in Dp1Tyb and Dp3Tyb hearts, suggesting that impaired proliferation may also play an important role in CHD. Changes in several other pathways, such as increased expression of inflammatory, interferon response and EMT genes, were also seen in the human and mouse embryonic hearts. Since changes in these pathways were seen in hearts from Ts1Rhr mice, they are not sufficient on their own to cause heart defects. However, they may also contribute to CHD pathology, in combination with the mitochondrial and proliferative deficits.

It remains unclear how defects in mitochondrial function and proliferation in most cardiac cells can cause localized defects in septation, rather than a broader cardiomyopathy. One possibility is that the cellular changes are relatively small and that they preferentially affect structures involved in septation such as the ventricular septum, the atrioventricular and outflow tract cushions or the dorsal mesenchymal protrusion. Further work is needed to understand the developmental defects in Dp1Tyb mouse hearts that lead to VSD and AVSD.

Mitochondrial dysfunction has been previously reported in DS (27–30). Human DS fibroblasts, astrocytes and neurons have impaired mitochondrial respiratory activity, decreased mitochondrial membrane potential and altered mitochondrial shape with smaller and more fragmented mitochondria (31–34). This dysfunction may underlie the neurological and cognitive impairment in DS, contributing to decreased neurogenesis and altered processing of APP, leading to deposition of Aβ amyloid and early-onset Alzheimer's disease. Our results show that a very similar mitochondrial phenotype is evident in embryonic cardiomyocytes from the Dp1Tyb mouse model of DS and suggest that mitochondrial dysfunction may play an important role in the cardiac pathology in DS. This commonality of mitochondrial impairment across multiple DS tissues has led to the interesting proposal that drugs that increase mitochondrial biogenesis or respiratory capacity may be promising therapeutic candidates for DS phenotypes, for example, for cognitive deficits (28–30). Our results suggest that a similar approach may ameliorate cardiac defects.

We previously reported that Dp3Tyb mouse embryos have CHD, but Dp4Tyb, Dp5Tyb and Dp6Tyb mouse embryos do not, implying that there must be at least two causative genes whose increased dosage leads to CHD (8). Since we have now shown that the Dp4Tyb region is not required for CHD (Fig. 5D), the causative genes must lie in the regions duplicated in Dp5Tyb and Dp6Tyb mice, with at least one causative gene in each region (the 2-locus hypothesis, fig. S6). Whereas in this study we have identified *Dyrk1a* as a causative gene within the Dp5Tyb region, the second causative gene, referred to as *GeneX*, is likely to be one of the six coding genes present in three copies in Dp3Tyb and Dp6Tyb mice but not Ts1Rhr mice (*Mx2, Tmprss2, Ripk4, Prdm15, C2cd2* and *Zbtb21*). None of these six genes has been previously implicated in the etiology of CHD. However, a study of DS individuals found that congenital hearts defects were associated with two short copy number variants located between *RIPK4* and *PRDM15* and within *ZBTB21* respectively, implicating this region in DS-CHD (35). In addition, a recent study of rare copy number variants associated with AVSD in the non-DS population identified several patients with an extra copy of regions of Hsa21 ranging from 10-21 Mb and spanning both regions that we have identified in this mouse study, and proposed *DYRK1A* as a candidate gene for CHD (36).

Increased dosage of *Dyrk1a* is necessary but not sufficient to cause the mitochondrial defects and CHD, since these are not seen in Ts1Rhr mouse embryonic hearts despite 3 copies of *Dyrk1a*. It is unclear how elevated DYRK1A activity, acting with the unknown other causative gene(s), causes mitochondrial changes and CHD. One possibility is that increased DYRK1A inhibits the function of NFAT transcription factors, since DYRK1A phosphorylates NFAT proteins leading to their nuclear exclusion (37), and mouse embryos deficient in both NFATc3 and NFATc4 have impaired mitochondrial function and defective cardiac development (38). Indeed, DYRK1A in collaboration with RCAN1, another Hsa21-encoded gene, negatively regulates NFAT proteins and overexpression of both genes in the developing mouse leads to failure of outflow tract valve elongation (37). Although these are not the same defects as seen in DS, which are usually defects in septation, it supports the view that DYRK1A, acting through NFAT proteins can perturb cardiac development. Alternatively, DYRK1A may affect mitochondrial function through the SIRT1 protein deacetylase, since DYRK1A binds to, phosphorylates and activates SIRT1 and overexpression of SIRT1 in cardiomyocytes impairs their mitochondrial respiration (39, 40). SIRT1 in turn may be acting through PGC1α (PPARGC1A), a regulator of mitochondrial biogenesis (41, 42).

Increased *Dyrk1a* dosage is also necessary for the impaired proliferation, potentially by phosphorylating Cyclin D proteins leading to their degradation. This would result in reduced CDK4/6 activity, decreased phosphorylation of RB1, reduced activity of E2F transcription factors and less expression of E2F target genes which are required for G1 to S phase transition. Our study supports such a mechanism. In agreement with this, overexpression of DYRK1A in the cardiomyocytes of adult mice leads to reduced amounts of CCND2, RB1 phosphorylation and RB1/E2F signaling and hence to impaired proliferation (20). Conversely, inhibition of DYRK1A or cardiomyocyte-specific deletion of the *Dyrk1a* gene leads to increased cardiomyocyte proliferation and cardiac hypertrophy in adult mice (43). DYRK1A has been shown to phosphorylate LIN52, leading to assembly of the repressive form of the DREAM complex which promotes entry into quiescence, providing another mechanism by which DYRK1A overexpression may regulate proliferation (44, 45).

DYRK1A phosphorylates many proteins (46, 47). For example, in addition to the substrates described above (NFAT, SIRT1, CCND2 and LIN52), DYRK1A phosphorylates the C-terminal domain of RNA polymerase II, Alternative Splicing Factor (ASF) and CAS9, thereby regulating transcription, splicing and apoptosis (48–51). Further studies are needed to determine whether any of these DYRK1A targets is involved in DYRK1A-induced CHD.

It has been proposed that the increased expression of interferon response genes in DS is caused by a third copy of four interferon receptor genes located on Hsa21 (52). Since these expression changes are also seen in Dp3Tyb and Ts1Rhr mouse hearts which do not have an extra copy of the interferon receptor genes, our results show that one or more of the 31 genes present in three copies in Ts1Rhr mice can also cause these inflammatory gene expression changes. A recent report has shown that a third copy of the interferon receptor genes causes CHD in DS (53). However, since Dp3Tyb mice that do not have an increased dosage of these genes show CHD (8), and Dp1Tyb*Dyrk1a*^+/+/-^ mice that still have three copies of the genes do not show CHD (this study), our results imply that increased dosage of the interferon receptor genes is neither necessary nor sufficient to cause CHD. Nonetheless it is possible that cardiac pathology results from a complex genetic interplay between *Dyrk1a*, the interferon receptor genes and other unknown causative genes.

This study has several limitations. It is unclear why Leucettinib-21 does not have a stronger effect on transcriptional changes in Dp1Tyb embryos, but possibilities include limited access of Leucettinib-21 to embryonic cardiac cells and pharmacokinetic issues such as a short half-life of the inhibitor in the embryo. Further work is needed to evaluate these issues. The work presented here shows that mitochondrial dysfunction and reduced cell proliferation correlate with congenital heart defects and that all three of these depend on a third copy of *Dyrk1a*, however the results do not prove that these cellular changes cause septation defects. Finally, the congenital heart defects are caused by increased dosage of at least two genes. In this study we have shown that one of these is *Dyrk1a*. Again, further work will be needed to identify the second causative gene.

We propose that CHD in DS arises in part from increased DYRK1A activity in cardiomyocytes leading to reduced proliferation and mitochondrial dysfunction (Fig. 8E). Furthermore, our systematic genetic mapping approach for dosage-sensitive genes can be used to identify causative genes and mechanisms responsible for the many other phenotypes of DS.

## Materials and Methods

### Study design

The aim of this study was to identify the genes that are necessary in three copies to cause congenital heart defects in DS, and to investigate how such increased dosage contributes to pathology. The study used the Dp1Tyb mouse model of DS in which embryos have heart defects similar to those seen in human DS. In addition, we also used tissue from human fetal hearts with DS, as well as euploid controls. Frozen human fetal hearts (13-14 pcw, 5 DS and 5 euploid age- and sex-matched samples) were obtained from the MRC-Wellcome Trust Human Developmental Biology Resource (HDBR) under appropriate ethical approval from the local Research Ethics Committees. UCL site REC reference: 18/LO/0822 – IRAS project ID: 244235 and Newcastle site REC reference: 18/NE/0290 – IRAS project ID: 250012. RNAseq was used to investigate transcriptional changes in human DS fetal hearts and in Dp1Tyb mouse embryonic hearts. Mitochondrial function and cell proliferation were measured in the mouse embryonic hearts. We used mouse genetics to identify one of the causative genes, *Dyrk1a*, and tested whether three copies of this gene were necessary to cause the transcriptional changes in Dp1Tyb embryonic hearts, as well as the changes in mitochondrial function and cell proliferation. Sample sizes were selected based on previous experience with similar methods. No randomization was performed. Investigators were blinded to genotype when scoring mouse embryonic hearts for the presence of heart defects. Human samples (DS and euploid) were balanced for age and sex. Mouse embryos were age-matched and mutant and control embryos were littermates. No samples were excluded from the study. Details on samples sizes representing biological replicates and statistical tests are detailed in figure legends and in the Statistical Analysis section of the Materials and Methods. Data are provided in Data file S1.

### Mice

Mice carrying the following alleles have been described previously: Dp(16Lipi-Zbtb21)1TybEmcf (Dp1Tyb), Dp(16Mir802-Zbtb21)3TybEmcf (Dp3Tyb), Dp(16Cbr1-Fam3b)1Rhr (Ts1Rhr), *Kcnj6*^tm1Stf^ (*Kcnj6*^-^), *Dyrk1a*^tm1Mla^ (*Dyrk1a*^-^), and *Dyrk1a*^tm2Yah^ (*Dyrk1a*^K188R^) (8, 25, 55–57). Dp1Tyb mice have been deposited with JAX (strain #037183). Mice with the Del(16Mir802-Vps26c)4TybEmcf (Del4Tyb) allele were generated using an in vivo Cre-mediated recombination strategy by breeding female mice containing the *Hprt*^tm1(cre)Mnn^ allele (58) and two loxP sites located in trans configuration on Mmu16 at the boundaries of the desired deletion, to C57BL/6J males. Cre activity in the female germline from the *Hprt*^tm1(cre)Mnn^ allele resulted in occasional pups (5.3%) with recombination between the loxP sites generating the Del4Tyb deletion. Mice carrying the loxP sites were derived from targeting ES cells with MICER vectors MHPN219l02 (16:92850909 – 16:92859345 Mb, coordinates from mouse genome assembly GRCm39), located between *Runx1* and *Mir802*, and MHPP432c09 (16:94339475 – 16:94347709 Mb), located between *Vps26c* and *Dyrk1a* as previously described (8). The integrity of the Del4Tyb mutation was validated by comparative genome hybridization (fig. S4A). ES cells carrying the *Setd4*^tm1a(KOMP)Wtsi^ allele (59) were obtained from the International Knockout Mouse Consortium and used to establish a mouse strain which was bred first to Tg(CAG-Flpo)1Afst mice (60) which express Flp in the germline to delete LacZ and Neo genes, generating mice with the *Setd4*^tm1c(KOMP)Wtsi^ (*Setd4*^fl^) allele in which exon 6 (ENSMUSE00001268769) is flanked by loxP sites. These in turn were bred to Tg(Prm-cre)70Og mice (61) expressing Cre in the male germline to generate mice bearing the *Setd4*^tm1d(KOMP)Wtsi^ (*Setd4*^-^) allele in which exon 6 had been deleted. Mice with the *Mir802*^em1Tyb^ (*Mir802*^-^) allele were generated by direct injection of Cas9 and 2 guide RNAs (5’-TCTACATAACCTACCGACTGCGG-3’ and 5’-ACGCCCTCCGAGGACACCCCAGG-3’) into mouse zygotes to generate a 141 bp deletion (16:93166602 - 16:93166742) of the *Mir802* gene. Since both Dp1Tyb and *Dyrk1a*^+/-^ mice are poor breeders, we found it impossible to breed sufficient numbers of Dp1Tyb*Dyrk1a*^+/+/-^ mice (nomenclature indicates mice with two WT alleles of *Dyrk1a* and one deleted allele) by simply intercrossing these strains. To overcome this, rare mice bearing both the Dp1Tyb and *Dyrk1a*^-^ alleles were crossed to C57BL/6J mice and a pup was identified where a crossover had brought the two alleles onto the same chromosome. The resulting Dp1Tyb*Dyrk1a*^+/+/-^ mice bred well and were maintained as a separate strain, with WT littermate embryos used as controls. RNAseq analysis confirmed that one *Dyrk1a* allele had a deletion of exons 7 and 8 (fig. S4B). All mice were bred and maintained on a C57BL/6J background (backcrossed for ≥ 10 generations), initially at the MRC National Institute for Medical Research and then at the Francis Crick Institute. All animal experiments were carried out under the authority of a Project Licence granted by the UK Home Office, and were approved by the Animal Welfare Ethical Review Body of the Francis Crick Institute. Numbers of protein-coding genes in different mouse strains were determined using the Biomart function in Ensembl on mouse genome assembly GRCm39, filtering for protein-coding genes, excluding three genes: ENSMUSG00000116933 which is a partial transcript for *Atp5o* (ENSMUSG00000022956), *Gm49711*, which is an alternatively spliced form of *Mrps6*, and *Gm49948* which is a fusion transcript of some exons from *Igsf5* and *Pcp4*. Note that the numbers of duplicated coding genes in the Dp strains have changed since our original publication (8), due to changes in gene annotation.

### Statistical analysis

Statistical analysis of differential gene expression in RNAseq data was performed using the DESeq2 package. The significance threshold for the identification of differentially expressed genes, corrected for multiple testing, was set as an adjusted *P*-value <0.05. Gene set enrichment analysis was carried out with the GSEA software, calculating a normalized enrichment score and a false discovery rate (FDR). An FDR <0.05 was considered significant. Statistical analysis of proteomic data was carried out using a Welch t-test to evaluate the significance of differences between samples of the two genotypes, generating an FDR corrected *P*-value; a *P*-value of <0.05 was considered significant. Other data were analyzed using Fisher's exact test, a two-sided Mann Whitney test or Kruskal-Wallis test as detailed in figure legends. A *P*-value of <0.05 was considered significant.

## Supplementary Material

Data file S1

Table S1

Table S2

Table S3

Table S4

Table S5

Table S6

Table S7

Table S8

Table S9

Table S10

Supplementary Materials and Methods

## Data Availability

All data associated with this study are present in the paper or supplementary materials. Dp1Tyb mice are available from JAX (strain #037183). Dp1Tyb and Dp3Tyb mice are available from the European Mouse Mutant Archive. Mice carrying the Del4Tyb, *Setd4*^tm1d(KOMP)Wtsi^ and *Mir802*^em1Tyb^ alleles are available on request to the corresponding author under an MTA. All bulk and single-cell RNAseq data have been deposited in the Gene Expression Omnibus, accession code: GSE196447. Mass spectrometry proteomic data have been deposited in the ProteomeXchange through the PRIDE partner repository, dataset identifier: PXD013053.

## One Sentence Summary

Increased DYRK1A dosage in the Dp1Tyb mouse model of Down syndrome causes mitochondrial dysfunction and congenital heart defects.
