# Supplementary material for: Increased dosage of DYRK1A leads to congenital heart defects in a mouse model of Down syndrome
Source: Sci Transl Med. Author manuscript; Available in PMC 2024 Feb 16. (PMC7615651; doi:10.1126/scitranslmed.add6883)
Supplement: Supplementary Materials and Methods [file EMS193971-supplement-Supplementary_Materials_and_Methods.pdf]

Increased dosage of DYRK1A leads to congenital heart defects in a mouse model of Down syndrome

Supplementary Materials and Methods

Array Comparative Genome Hybridization (aCGH)

Genomic DNA was prepared from tails of Del4Tyb (test) and C57BL/6J (control) mice. DNA, labeled with Cy3 (test) and Cy5 (control), was hybridized by Roche Diagnostics Limited to a mouse 3x720K array (Roche NimbleGen) containing 50-75mer probes designed based on mouse genome assembly MGSCv37. The hybridized aCGH slides were scanned in the Cy3 and Cy5 channels. The Log2 ratios of the test/control signals were calculated for all probes and the ratios for the probes on Mmu16 were plotted in genomic order to visualize the deleted region.

RNA sequencing (RNAseq)

Frozen human fetal hearts (13-14 pcw, 5 DS and 5 euploid age- and sex-matched samples) were obtained from the MRC-Wellcome Trust Human Developmental Biology Resource (HDBR). Frozen tissues were placed in RLT lysis buffer (Qiagen) in gentleMACS M-tubes (Miltenyi Biotec), homogenized using the gentleMACS Octo dissociator (Miltenyi Biotec) and RNA was extracted using the RNAeasy Maxi kit (Qiagen). RNA integrity numbers (RIN) of the samples were 7-9.4. Stranded polyA-enriched libraries were made using the KAPA mRNA HyperPrep kit (Roche) according to the manufacturer's instructions and sequenced on the HiSeq 4000 (Illumina) with single-ended 100 base reads. An average of 34 million reads were generated per sample.

E13.5 mouse hearts from Dp1Tyb, Dp1Tyb*Dyrk1a*$^{+/+/-}$, Dp3Tyb and Ts1Rhr embryos and WT littermate embryos for each strain (5 per genotype) were dissected and snap frozen. Frozen tissues were placed in lysis buffer and homogenized using a cordless Pellet pestle (Sigma-Aldrich) followed by RNA extraction using RNAeasy mini kit (Qiagen). RNA integrity numbers (RIN) of the samples were 9.8-10. Stranded polyA-enriched libraries were made using the KAPA mRNA HyperPrep kit (Roche) according to the manufacturer's instructions and sequenced on the HiSeq 2500 or HiSeq 4000 (Illumina) with single ended 100 base reads. An average of 24 million reads were generated for the Dp1Tyb and Dp1Tyb*Dyrk1a*$^{+/+/-}$ and WT samples and an average of 34 million reads for Dp3Tyb, Ts1Rhr and corresponding WT samples. Adaptor trimming was performed with Trimmomatic/0.36-Java-1.7.0_80 with parameters "LEADING:3 TRAILING:3 SLIDINGWINDOW:4:20 MINLEN:36" (*62*). The RSEM package (v.1.2.31) in conjunction with the STAR alignment algorithm (v.2.5.2a) was used for the read mapping and gene-level quantification with respect to mouse genome assembly GRCm38 (release 86) for the mouse data sets and human genome assembly GRCh38 (release 89) for the human samples (*63, 64*). Unsupervised hierarchical clustering was carried out using Euclidean distance with the heatmap.2 function from gplots (*65*). Differential expression analysis was performed with the DESeq2 package within the R programming environment (*66, 67*). The significance threshold for the identification of differentially expressed genes was set as an adjusted *P* value ≤0.05. Gene set enrichment analysis was carried out with the GSEA software (version 2.2.3) (*10*). The software compares ranked lists of genes, in this case, differentially expressed genes ranked by the "stat" value in decreasing order, with the following gene sets from the Molecular Signature Database: "c2.cp.v7.0.symbols.gmt", "c5.bp.v7.0.symbols.gmt", "h.all.v7.0.symbols.gmt" downloaded from the Broad Institute (http://www.broad.mit.edu/gsea/). GSEAPreranked was used with "classic" enrichment analysis not excluding large datasets.

For the pharmacological rescue experiment, 5 E13.5 hearts were analyzed for each of the 4 conditions: 2 genotypes (WT and Dp1Tyb) x 2 treatments (LCTB-21 and iso-LCTB-21). Both male and female embryos were analyzed, with the same numbers of each sex in each of the 4 groups. Samples were dissected and snap frozen. Frozen tissues were placed in lysis buffer and homogenized using a cordless Pellet pestle (Sigma-Aldrich) followed by RNA extraction using RNAeasy micro kit (Qiagen). RNA integrity numbers (RIN) of the samples were 10. Stranded polyA enriched libraries were generated using NEBNext Ultra II Directional RNA Library Prep Kit for Illumina according to manufacturer's instructions. An average of 25 million paired end reads per library (PE100) were generated on a NovaSeq6000 (Illumina). All samples were processed using the nf-core RNAseq pipeline (v.3.10.1) operating on Nextflow (v.22.10.3) (*68, 69*). Settings for individual tools were left as standard for this workflow version unless otherwise specified. Alignment to GRCm38 (release 95) was performed using STAR (v2.7.10a) (*64*), followed by quantification using RSEM (v1.3.1) (*63*). Results were further processed in R (version 4.2.0) (*67*). Outputs of the RNAseq pipeline were assessed for quality using the metrics provided by the pipeline's inbuilt quality control packages, PCA (package stats v4.2.0), and correlation analyses between samples (package stats v4.2.0). Differential expression analysis was performed with DESeq2 (v1.38.3) (*66*), using a model accounting for differences in litter, sex, treatment and genotype (~ *litter* + *sex* + *treatment* * *genotype*). The significance threshold for the identification of differentially expressed genes was set as an adjusted *P* value ≤0.05, where *P* values were adjusted for false discovery rates according to the Independent Hypothesis Weighting method (package IHW, v1.26.0) (*70, 71*). Bulk RNAseq data have been deposited in the Gene Expression Omnibus, accession codes: GSE196447 and GSE239798.

Single cell RNAseq

E13.5 mouse hearts from Dp1Tyb, Dp1Tyb*Dyrk1a*$^{+/+/-}$ and WT littermate embryos were dissected and subsequently dissociated into single cells using the Neonatal Heart Dissociation kit and gentleMACS Octo dissociator (Miltenyi Biotec). Cells in suspension were methanol fixed, filtered through a 30 µm cell strainer, spun down and stored at -20ºC. Fixed cells were resuspended in PBS, 0.04% BSA, assessed for viability using trypan blue staining and quantified using the EVE automated cell counter (Cambridge Bioscience). Cells were processed using the Chromium 3' mRNA-Seq version 2 kit (10x Genomics). Samples were sequenced on the HiSeq 4000 acquiring at least 142,000 reads per cell, achieving a sequencing saturation of 73 - 90%.

Cell Ranger software (version 2.1.1, 10x Genomics) was used to de-multiplex Illumina BCL output, create fastq files and generate single cell feature counts for each library using GRCm38 (v1.2.0) as reference. All subsequent analyses were performed in R (v3.6.1) using the Seurat (v 3.1) package (*67, 72*). Genes were removed if they were expressed in 3 or fewer cells and cells with < 200 expressed genes detected were also removed. After quality control and filtering 3,384 cells were retained for further analysis. Data was integrated following Seurat's vignette. In brief, for each sample the top 2000 most variable genes were selected for data integration using Canonical correlation Analysis (CCA) with 50 dimensions for dimensional reduction using tSNE and cluster calling using the Louvain algorithm. Clusters were visualized using the Uniform Manifold Approximation and Projection (UMAP). Upon initial examination of the clusters, we detected small clusters corresponding to red blood cells and lung tissue, both of which were removed. Subsequently data was normalized and integrated, and clustering analysis was repeated with the same parameters as above, with the addition that the effects of cell cycle were regressed out. A cluster resolution of 0.4 was used to define clusters for further analysis.

Cluster markers were identified using "FindConservedMarkers" with default parameters. Differentially expressed genes within each cluster were identified using the DESeq2 test in "FindMarkers" using data in which the effects of cell cycle had not been regressed out. scRNAseq data have been deposited in the Gene Expression Omnibus, accession code: GSE196447.

Proteomics

E13.5 mouse hearts from Dp1Tyb*Dyrk1a*$^{+/+/-}$ and WT littermate embryos (5 per genotype) were dissected, snap frozen and stored at -80ºC. Frozen tissues were placed in 8M Urea with phosSTOP protease inhibitors (Roche) and homogenized using a cordless Pellet pestle (Sigma-Aldrich). Lysates were reduced, alkylated and digested with tryspin using standard methods. Nest Group C_{18} MacroSpin columns (SMMSS18V) were used to concentrate and clean up the peptides according to the manufacturer supplied instructions, prior to labeling with TMT10plex Isobaric Label Reagent Set (ThermoFisher Scientific). TMT-labeled samples were pooled, enriched for phospho-peptides using the High-Select Fe-NTA phosphopeptide enrichment kit (A32992, ThermoFisher Scientific) and using Titansphere TiO 5 μm bulk media (GL Sciences) according to the manufacturer supplied protocols. Samples were analyzed by LC-MS/MS on an Orbitrap Fusion Lumos Mass Spectrometer (ThermoFisher Scientific). Raw data were processed in MaxQuant v1.6.0.13 (https://www.maxquant.org/), with the database search conducted against the canonical sequences of the UniProt Mus Musculus complete proteome, downloaded August 2017. Statistical analysis was carried out using Perseus (https://www.maxquant.org/perseus/). In brief, abundance values for each protein were log2 transformed, median normalized and a Welch t-test was used to evaluate the significance of differences between samples of the two genotypes, generating an FDR corrected p-value and log2(fold change of the geometric means). The mass spectrometry

proteomics data have been deposited with the ProteomeXchange Consortium (http://proteomecentral.proteomexchange.org) through the PRIDE partner repository (*73*), dataset identifier PXD013053.

HREM imaging and 3D modeling

E14.5 embryonic hearts were dissected and fixed for 30 min in 4% paraformaldehyde followed by a 1 h wash in distilled water and fixed a second time overnight. Fixed samples were dehydrated and embedded in modified JB4 methacrylate resin (*74*). Samples were sectioned at 2.5 µm and imaged using a Jenoptik camera with an isometric resolution of 2 µm. Data sets were normalized and subsampled prior to 3D volume rendering using OsiriX MD (*75*). Phenotype analysis was performed blind for genotype and classification of type of CHD was carried out as previously described (*76*). Mutant and control WT hearts were taken from the same litters.

Flow cytometry

E13.5 mouse hearts from Dp1Tyb and WT littermate embryos and Dp1Tyb*Dyrk1a*$^{+/+/-}$ and their WT littermates were dissociated into single cells using the Neonatal Heart Dissociation kit and gentleMACS Octo dissociator (Miltenyi Biotec). To evaluate mitochondrial membrane potential and content cells in suspension were incubated with 20 nM tetramethylrhodamine methyl ester (TMRM, Thermo Fisher) and 20 nM of Mitotracker Green (MTG, Thermo Fisher), respectively, for 30 min at 37°C. Cells were then stained with anti-CD106-APC, anti-CD31-BV785 and Zombie Aqua (all Biolegend, 105718, 102435, 423102, respectively). CD106 (VCAM-1) is predominantly expressed on cardiomyocytes in mouse embryonic hearts (*77*). CD31 (PECAM1) is expressed on both endocardial and endothelial cells, but in E13.5 hearts most CD31^{+} cells are endocardial cells (Figure 2C). Zombie Aqua was used to distinguish live and dead cells. To analyze

cell cycle phases, dissected E13.5 mouse hearts were incubated with 10 μM EdU at 37°C for 30 min, and then dissociated into single cells using the Neonatal Heart Dissociation kit and gentleMACS Octo dissociator (Miltenyi Biotec). Cells in suspension were stained in PBS with anti-CD106-APC, anti-CD31-BV785 (Biolegend) and Live/dead Near-IR (ThermoFisher, L10119) for 30 min, followed by fixation in 4% Paraformaldehyde (PFA) for 20 min. EdU was detected using the Click-it EdU kit (Life Technologies), and DNA was stained with FxCycle violet (ThermoFisher). Data were acquired on an LSR Fortessa X-20 cell analyzer (BD) and analyzed using FlowJo v9.

Immunoblot analysis

E13.5 mouse hearts from Dp1Tyb and WT littermate embryos were dissected and snap frozen. Frozen tissues were placed in RIPA lysis buffer and homogenized using a cordless Pellet pestle (Sigma-Aldrich) followed by protein extraction. Total protein lysates (20 μg) were resolved on a denaturing 4-10% precast SDS-PAGE (Bio-Rad) and probed with rabbit monoclonal antibodies against phospho-RB1 (Ser807/Ser811) and GAPDH (Cell Signaling Technology, 8516 and 5174, respectively). Binding of primary antibodies was detected using AF680–conjugated anti-rabbit IgG (Thermo Fisher Scientific). Fluorescence from the secondary reagents was detected using an Odyssey (LI-COR Biosciences). For quantitation, signal from phospho-RB1 was normalized to GAPDH.

Metabolic analysis

E13.5 mouse hearts from Dp1Tyb and WT littermate embryos and Dp1Tyb*Dyrk1a*$^{+/+/-}$ and their WT littermates were dissociated into single cells as described above. To measure rates of mitochondrial respiration, cells were seeded onto Seahorse 8-well plates in in XF D-MEM Medium pH 7.4 containing 10 mM glucose, 2 mM L-glutamine and 1 mM sodium pyruvate (all Agilent Technologies). The plates were centrifuged at 100xg for 3 min, kept

at 37°C for 45-60 min and the oxygen consumption rate (OCR) was measured using a Seahorse XFp Analyzer (Agilent Technologies). To investigate mitochondrial respiration phenotypes, a Seahorse XFp Cell Mito Stress Test kit (Agilent Technologies) was used. Cells were analyzed for 20 min and then oligomycin (1 μM, final concentration) was added, followed by carbonyl cyanide 4-(trifluoromethoxy) phenylhydrazone (FCCP) (1 μM, final concentration) 20 min later, and rotenone and antimycin A (0.5 μM, final concentration) a further 20 min after that. To measure glycolysis, cells were seeded onto Seahorse 96-well plates in in XF D-MEM Medium pH 7.4 containing 2 mM L-glutamine. The plates were centrifuged at 100xg for 3 min, kept at 37°C for 45-60 min and the extra-cellular acidification rate (ECAR) was measured using a Seahorse XFe96 Analyzer, using a Seahorse XFe96 Glycolysis stress Test kit (Agilent Technologies). To establish the basal ECAR, ECAR was measured for 20 min at the beginning of the assay. After 20 min, glucose (10 mM) was added, followed by oligomycin (1 μM) 20 min later, and 2-deoxy-D-glucose (50 mM) a further 20 min after that. Cells were fixed with 4% PFA for 15 min, nuclei were stained with 4′,6-diamidino-2-phenylindole (DAPI) and imaged using an EVOS microscope (Thermo Fisher). The Analyze Particles command from ImageJ was used to calculate numbers of nuclei which were used to normalize the OCR and ECAR data to numbers of cells in a well. Further analysis of OCR and ECAR was carried out using WAVE (version 2.6.1, Agilent Technologies).

Mitochondrial shape analysis

E13.5 mouse hearts from Dp1Tyb and WT littermate embryos and Dp1Tyb*Dyrk1a*$^{+/+/-}$ and their WT littermates were dissociated as previously described. Cells were seeded on 96-well plates (Greiner Bio-One) and cultured overnight to allow them to adhere. Mitochondria were stained by incubating the cells with 20 nM of MitoTracker Deep Red (MTDR) for 30 min at 37°C and fixed with 2% PFA for 20 min at 37°C. Cells were stained with rat anti-

mouse CD106 (BD Bioscience) overnight at 4°C, followed by goat anti-rat IgG Alexa Fluor 488 (Thermo Fisher) for 1 h at room temperature, and DAPI for 10 min at room temperature. Cells were imaged with the Opera Phenix High-Content Screening System (PerkinElmer). Initially, images were acquired using PreciScan imaging with a x20/NA 0.4 air lens and the locations of single cardiomyocytes ($CD106^+$) were defined using Harmony software V4.9. Next, single cardiomyocytes were re-imaged using a x63/NA 1.15 water-immersion lens. Z-stacks from 0 to 3 µm with a step size of 1 µm were acquired using excitation lasers at 405 nm (DAPI), 488 nm (anti-CD106) and 640 nm (MTDR). The mitochondrial network was identified using the MTDR signal (mitochondria) transformed into Ridge texture using the SER (Saddles, Edges, Ridges) feature of Harmony V4.9 (PerkinElmer). Mitochondrial shape was assessed using aspect ratio (major axis length/minor axis length), a measure of the length to width ratio, and form factor ($perimeter^2/4\pi[area]$), reflecting the complexity and branching of mitochondria (*54*). These parameters were determined using Harmony software on maximum projection images (fig. S3A, B).

Hypoxia analysis

E13.5 pregnant mice were injected i.p. with 60 mg/kg of Hypoxyprobe-1 (pimonidazole HCl, Hypoxyprobe, Inc.) in saline. Embryonic hearts were dissected 2 to 3 h post injection and fixed for 15 min in 4% paraformaldehyde followed by 1 h wash in distilled water and fixed a second time overnight. Hearts were paraffin-embedded and sectioned at 4 µm thickness and mounted on SuperFrost Plus slides. Hypoxyprobe was detected using a rabbit anti-pimonidazole antibody from the Hypoxyprobe Omni-Kit (Hypoxyprobe, Inc.), mouse anti-cardiac troponin (MA5-12960, Thermo Scientific) and rat anti-endomucin (Sc-65495, Santa Cruz) overnight at 4°C, followed by goat anti-rabbit IgG Cy3, goat anti-

mouse IgG AF647 and goat anti-rat IgG AF488 (Thermo Fisher) for 1 h at room temperature, and DAPI for 10 min at room temperature.

RNAscope

E12.5 mouse embryos and E14.5 dissected hearts were fixed in 10% neutral buffered formalin for 24 h, dehydrated, embedded in paraffin, and sectioned sagittally or frontally, respectively. Expression of *Dyrk1a* RNA on the sections was detected using ACD RNAscope 2.5 HD Assay - BROWN with a probe targeting the *Dyrk1a* gene (325-1455 bp, ACD catalogue number 432191) according to manufacturer's instructions. Slides were counterstained with haematoxylin.

Treatment of pregnant mice with Leucettinib-21

C57BL/6J female mice were mated with C57BL/6J males. From 5 days post coitum (E5.5), pregnant mice were orally gavaged daily with 0.3, 3 or 30 mg/kg of Leucettinib-21 (Figure S5A, Perha Pharmaceuticals) in 0.5% carboxymethylcellulose sodium salt (Sigma 9004-32-4) until E14.5. Embryos were dissected 2 h after the final gavage, snap frozen and then analyzed by mass spectrometry for the presence of Leucettinib-21 (Oncodesign Services). Alternatively, C57BL/6J female mice were mated with Dp1Tyb males and pregnant mice were treated with 30 mg/kg of Leucettinib-21 or iso-Leucettinib-21 in 0.5% carboxymethylcellulose daily by oral gavage from E5.5 to E13.5 (for RNAseq) or E14.5 (for HREM). Embryonic hearts were dissected 2h after the final gavage and processed for RNAseq and HREM imaging as described above.

Supplementary Figures

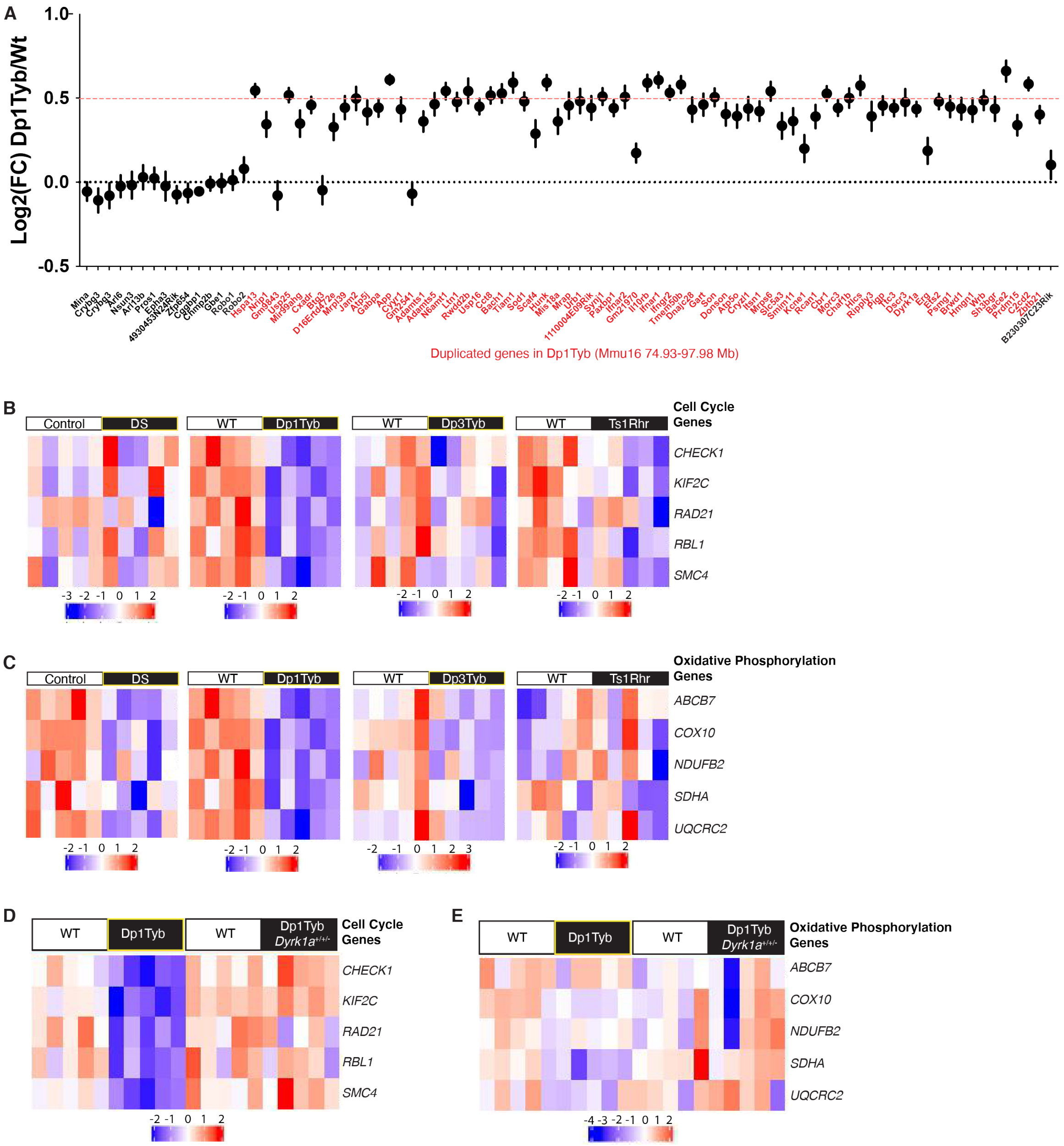

Figure S1. Altered gene expression in DS and Dp1Tyb embryonic hearts.

(**A**) Mean±SEM $\log_2$(fold-change) of gene expression between WT and Dp1Tyb E13.5 hearts (*n*=5). Only expressed genes are shown, defined as genes whose mean expression is >5 TPM and having a measured *P*-value in DEseq2. Genes within the duplicated region of Mmu16 are shown in red. For comparison, 15 genes that are centromeric to this region

of Mmu16 and one gene telomeric to this region and not duplicated are shown in black. Dashed red line indicates a fold change of 1.5 expected by the increased dosage of the duplicated genes; dotted black line represents no change in expression. All but three of the duplicated genes show increased expression. FC, fold-change. (**B-E**) Heatmaps showing change in gene expression of selected genes from (B, D) the Reactome cell cycle and (C, E) Hallmark oxidative phosphorylation gene sets in (B, C) DS and control euploid human embryonic hearts, and in Dp1Tyb, Dp3Tyb and Ts1Rhr mouse E13.5 hearts and their corresponding WT controls and (D, E) in Dp1Tyb and Dp1Tyb*Dyrk1a*$^{+/+/-}$ embryonic hearts and their corresponding WT littermate controls. Red and blue colors indicate increased or decreased expression of indicated genes relative to the mean expression of each row using normalized $\log_2$ expression values.

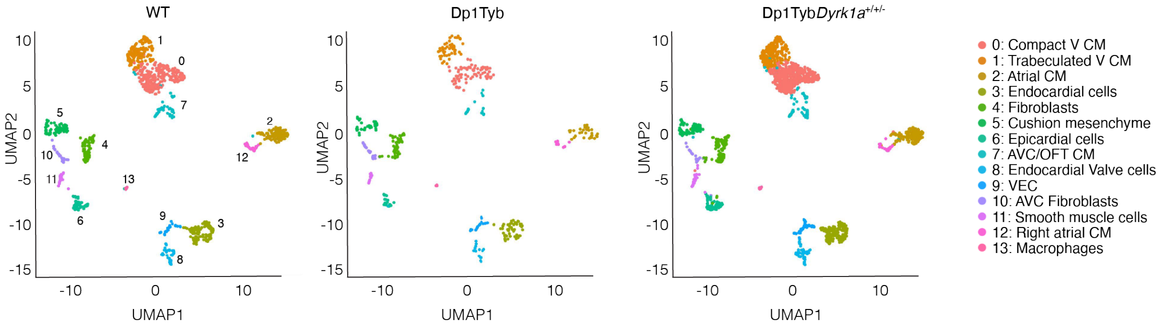

Figure S2. scRNAseq analysis of Dp1Tyb and Dp1Tyb *Dyrk1a*$^{+/+/-}$ embryonic hearts. UMAP plots of scRNAseq data from WT, Dp1Tyb and Dp1Tyb *Dyrk1a*$^{+/+/-}$ mouse E13.5 embryonic hearts. Clusters were generated by pooling data from all hearts and all genotypes as shown in Figure 2A. The plots in this figure use the same clustering but show cells in each genotype separately. Sample numbers: *n*=2 WT, 1 Dp1Tyb, 2 Dp1Tyb*Dyrk1a*$^{+/+/-}$.

A

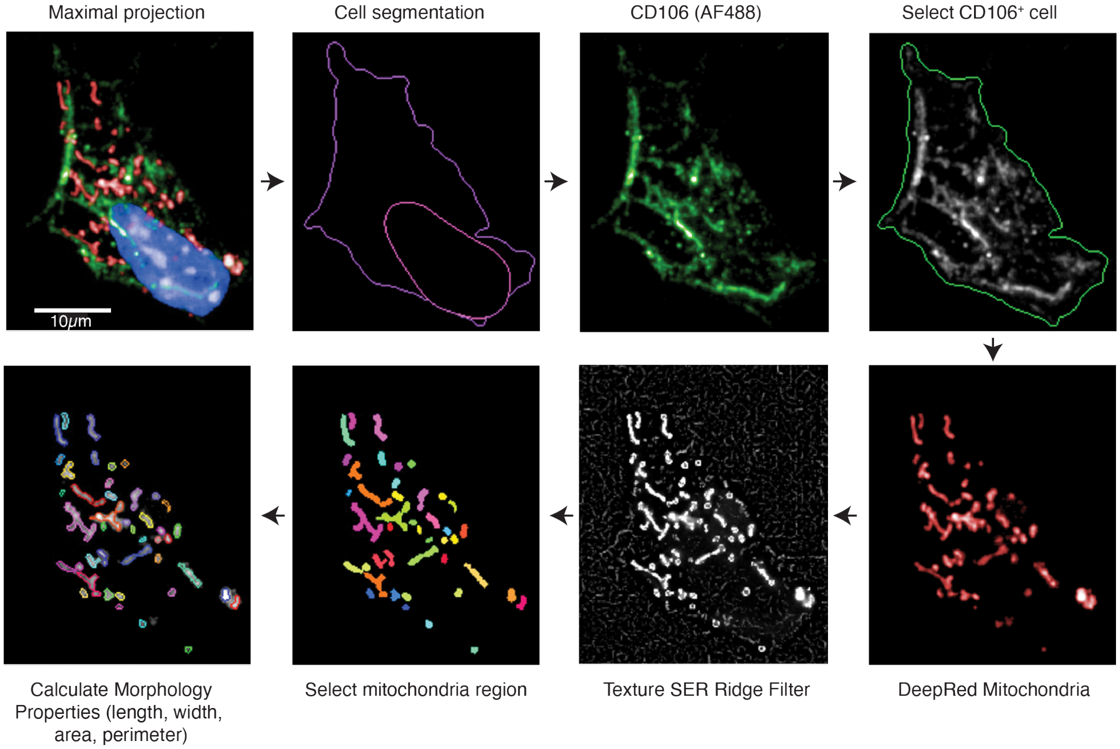

B

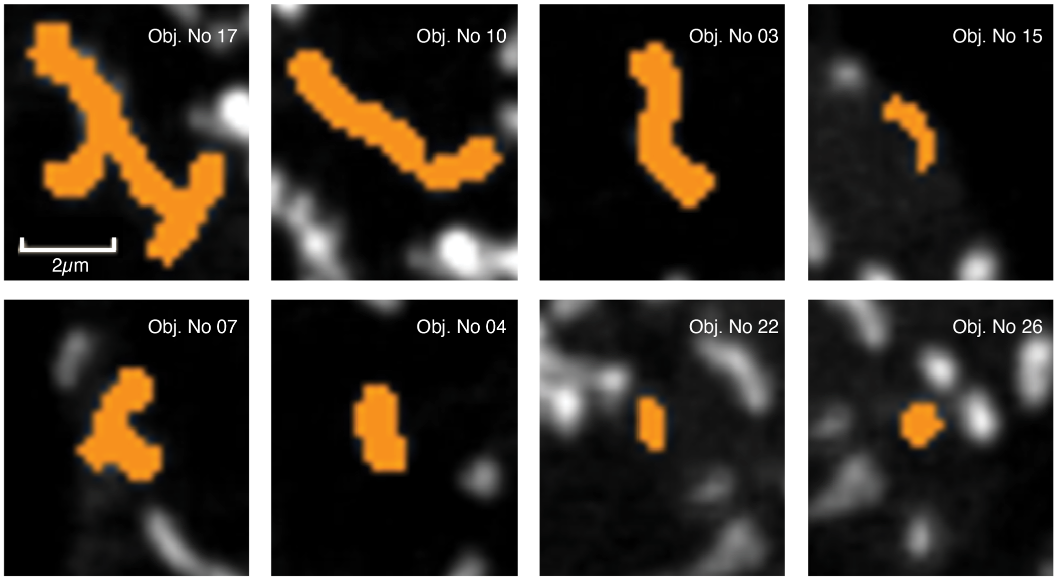

Object No	17	10	03	15	07	04	22	26
Form Factor	3.17	1.98	1.43	1.12	1.22	0.92	0.71	0.53
Aspect Ratio	6.90	6.48	4.67	3.02	2.70	2.20	1.90	1.03

Figure S3. Quantitation of mitochondrial morphology.

(**A**) Mouse embryonic heart single cells stained with MitoTracker Deep Red (MTDR), anti-CD106 (AF488) and DAPI were imaged by confocal microscopy. Maximal projection images (Z-stacks from 0 to 3 μm with a step size of 1 μm) were segmented to identify location of cells and nuclei. $CD106^+$ cells (cardiomyocytes) were selected for further analysis. The mitochondrial network was identified using the MTDR signal (mitochondria) transformed into Ridge texture using the SER (Saddles, Edges, Ridges) feature of Harmony. Mitochondrial network morphology was assessed by measuring the aspect ratio (major axis length/minor axis length) and form factor ($perimeter^2/4\pi[area]$), which are measures of distortion from circularity and degree of branching, respectively. (**B**) Eight example images of mitochondria (objects, Obj.) showing their aspect ratios and form factors.

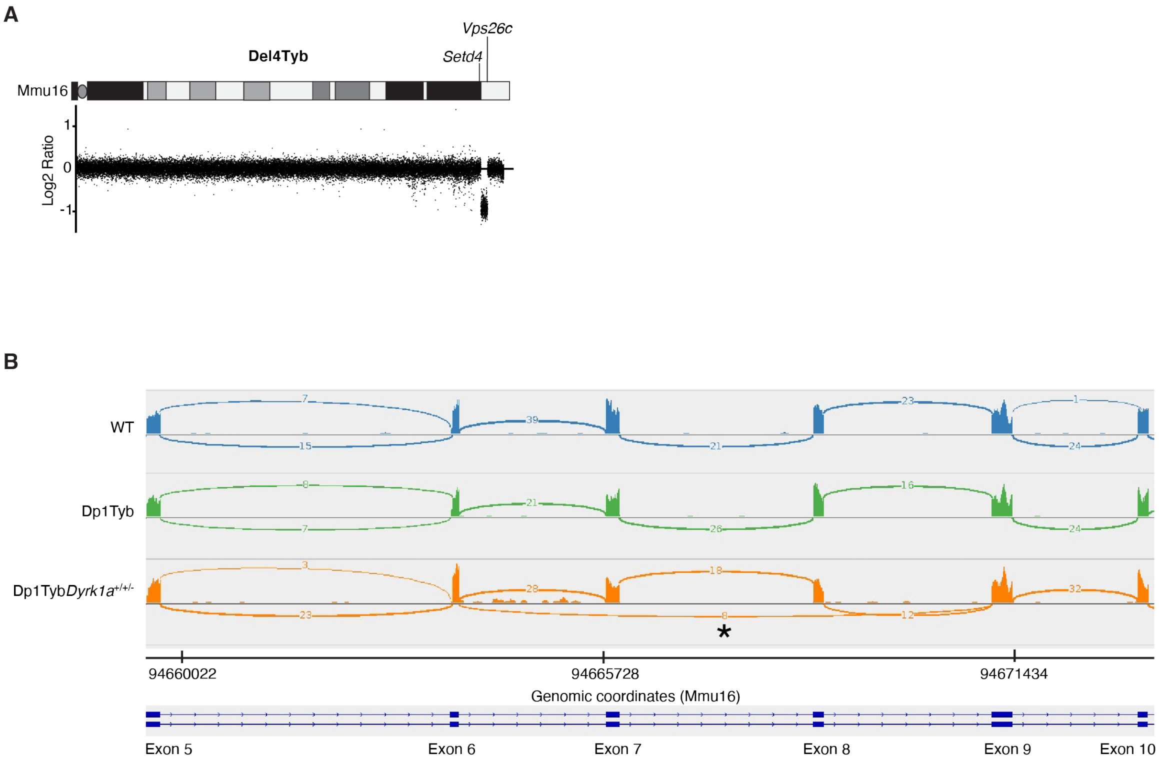

Figure S4. Validation of the Del4Tyb and Dp1Tyb*Dyrk1a*$^{+/+/-}$ mouse strains.

(**A**) Comparative genome hybridization analysis of the Del4Tyb mouse strain. Graph shows the $\log_2$(ratio) of the hybridization signal between Del4Tyb and C57BL/6J control mice. Each dot is a different probe along the length of Mmu16, in genomic order. Diagram at top shows a map of Mmu16 indicating the acrocentric centromere and the location of the *Setd4* and *Vps26c* genes marking the ends of the deletion. The deleted region is expected to have 0.5-fold decrease in DNA content, resulting in a $\log_2$(ratio) = -1. (**B**) Plot showing RNAseq reads mapped against the mouse genome within the *Dyrk1a* gene (exons 5 to 10) for E13.5 hearts from a WT, Dp1Tyb and Dp1Tyb*Dyrk1a*$^{+/+/-}$ embryo. Reads can be seen mapping against each exon and arcs indicate reads spanning introns. Numbers on the arcs show the numbers of reads showing the given splicing event. The Dp1Tyb*Dyrk1a*$^{+/+/-}$ embryo shows some reads connecting exon 6 to exon 9 (asterisk), as would be predicted if exons 7 and 8 had been deleted in one *Dyrk1a* allele. These

connecting reads are not seen in WT or Dp1Tyb mice, confirming successful deletion of exons 7 and 8 in Dp1Tyb*Dyrk1a*$^{+/+/-}$ embryos.

A

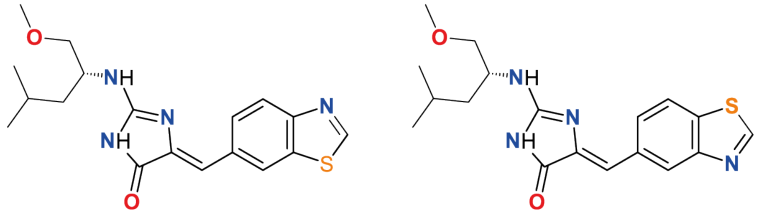

Leucettinib-21
IC_{50} (DYRK1A): 3.1 nM

iso-Leucettinib-21
IC_{50} (DYRK1A): >10 μM

B

Oral dose LCTB-21 (mg/kg)	LCTB-21 in embryo (ng/g)
0.3	ND
3	8.7±0.8
30	200.5±7.2

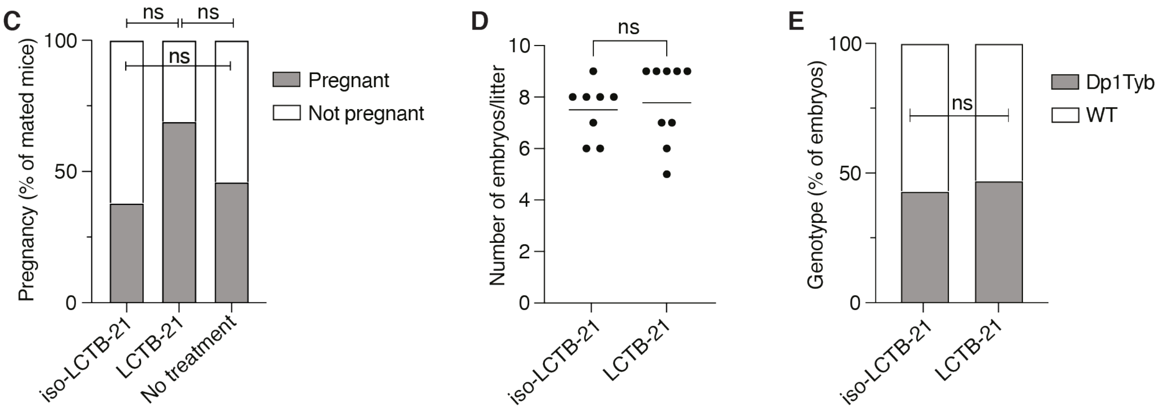

Figure S5. Treatment of pregnant mice with Leucettinib-21.

(A) Chemical structure of Leucettinib-21 (LCTB-21) and iso-Leucettinib-21 (iso-LCTB-21) indicating IC_{50} concentrations for the inhibition of DYRK1A in vitro (LCTB-21 is compound 106 in *24*). (**B**) Pregnant C57BL/6J mice were treated with the indicated dose of LCTB-21 daily by oral gavage from E5.5 to E14.5 and embryos analyzed for abundance of LCTB-21 by mass spectrometry, with values listed as mean (±SEM) ng of LCTB-21 per g of embryo tissue. ND, not detected. (**C-E**) C57BL/6J female mice that had successfully mated with Dp1Tyb male mice as judged by the presence of a vaginal plug on the day following mating (embryonic day 0.5, E0.5)

were treated daily with LCTB-21 or iso-LCTB-21 by oral gavage from E5.5 to E14.5 (Fig. 8A). Graphs show percentage of mated mice that (C) were pregnant at E14.5, (D) number of embryos per litter and (E) percentage of embryos whose genotype was WT or Dp1Tyb. Statistical analysis using Fishers exact (C, E) and Mann Whitney (D) tests; ns, not significant (P>0.05). Sample numbers: B, n=8 (0.3 or 3 mg/kg) and 4 (30 mg/kg) embryos; C, n=30 (iso-LCTB-21), 19 (LCTB-21) and 24 (no treatment) mice; D, n=8 (iso-LCTB-21) and 9 (LCTB-21) litters; E, n=60 (iso-LCTB-21) and 70 (LCTB-21) embryos.

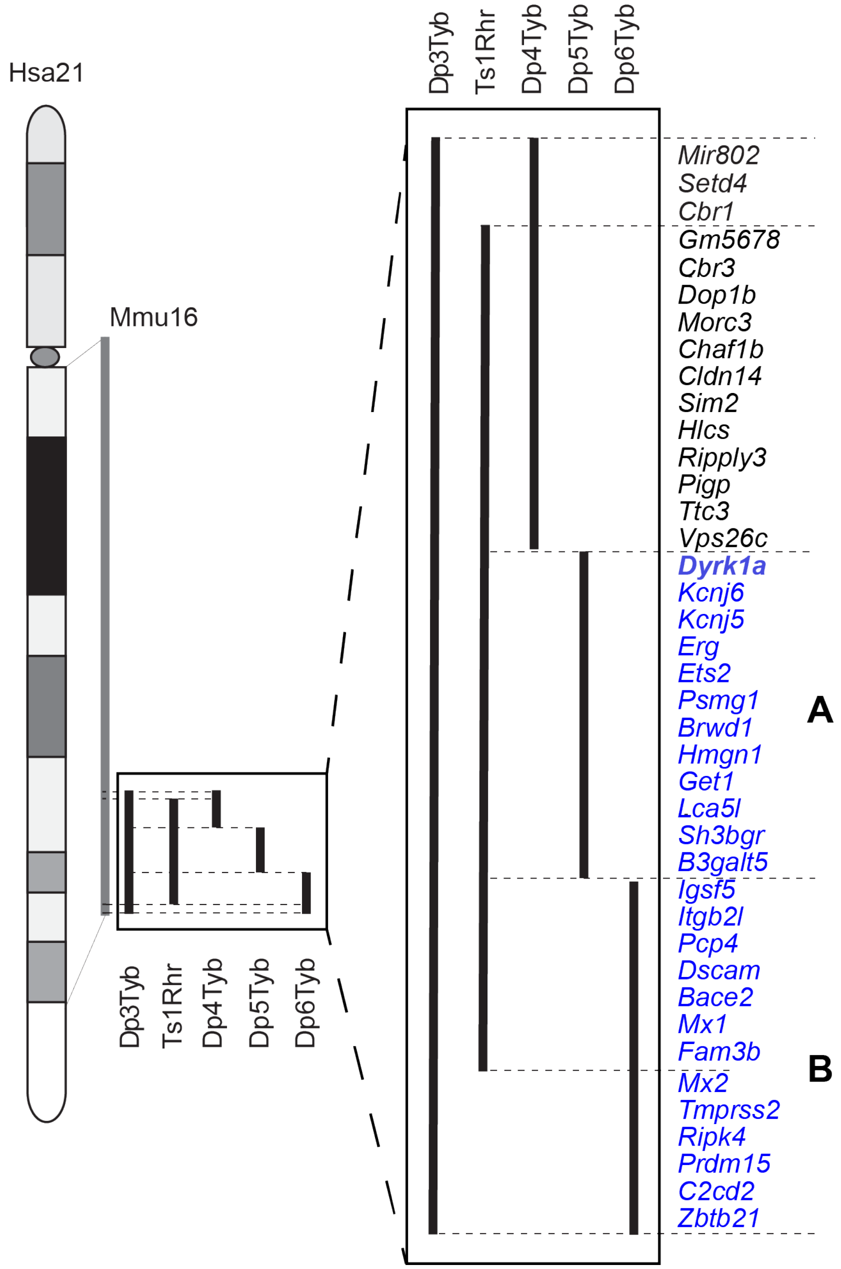

Figure S6. Regions of Hsa21 containing dosage-sensitive genes causing CHD.

Diagram of Hsa21 showing main cytogenic regions (rectangles of different shades) and the centromere (oval). Grey bar indicates the Hsa21-orthologous region of Mmu16. Black lines indicate regions of Mmu16 duplicated in the Dp3Tyb, Ts1Rhr, Dp4Tyb, Dp5Tyb and Dp6Tyb mouse strains. These regions are expanded showing all known protein coding genes within them and one microRNA gene (*Mir802*). Dp3Tyb mice have CHD, but

Ts1Rhr, Dp4Tyb, Dp5Tyb and Dp6Tyb do not, implying that there must be at least two causative genes. The genes in the Dp4Tyb region are not required for CHD, since a cross of Del4Tyb to Dp1Tyb did not affect the frequency of CHD. This implies that the two causative genes must lie in the regions duplicated in Dp5Tyb and Dp6Tyb respectively (regions A and B, genes in blue). We term this the 2-locus hypothesis. The *Dyrk1a* gene (bold) is required in three copies for the CHD phenotype, but there may be other genes in region A that are also required. The second unknown gene (*GeneX*) lies in region B and is most likely one of the 6 distal genes present in 3 copies in Dp3Tyb but not Ts1Rhr mice (*Mx2*-*Zbtb21*). Genes in regions that do not cause CHD are in black.

Supplementary Tables

Table S1. Human fetal hearts used for RNAseq analysis.

List of 10 human fetal heart samples obtained from HDBR (5 DS, 5 euploid) which were used for RNAseq showing gestational age and sex.

Table S2. RNAseq analysis of human Down Syndrome fetal hearts.

Sheet 1: Read me. Sheet 2: Expression of all genes in all samples in transcripts per million reads (TPM), and mean expression in each of the two genotypes (DS and euploid). Sheet 3: Differential gene expression analysis from DESeq2 showing for each gene its location, mean expression across all samples, log2(fold change) between DS and euploid and the *P*-value and adjusted *P*-value for this difference.

Table S3. RNAseq analysis of Dp1Tyb E13.5 mouse embryonic hearts.

Sheet 1: Read me. Sheet 2: Expression of all genes in all samples in transcripts per million reads (TPM), and mean expression in each of the two genotypes (Dp1Tyb and WT). Sheet 3: Differential gene expression analysis from DESeq2 showing for each gene its location, mean expression across all samples, log2(fold change) between Dp1Tyb and WT and the *P*-value and adjusted *P*-value for this difference.

Table S4. **RNAseq analysis of Dp3Tyb E13.5 mouse embryonic hearts.**

Sheet 1: Read me. Sheet 2: Expression of all genes in all samples in transcripts per million reads (TPM), and mean expression in each of the two genotypes (Dp3Tyb and WT). Sheet 3: Differential gene expression analysis from DESeq2 showing for each gene its location, mean expression across all samples, log2(fold change) between Dp3Tyb and WT and the *P*-value and adjusted *P*-value for this difference.

Table S5. RNAseq analysis of Ts1Rhr E13.5 mouse embryonic hearts.

Sheet 1: Read me. Sheet 2: Expression of all genes in all samples in transcripts per million reads (TPM), and mean expression in each of the two genotypes (Ts1Rhr and WT). Sheet 3: Differential gene expression analysis from DESeq2 showing for each gene its location, mean expression across all samples, log2(fold change) between Ts1Rhr and WT and the *P*-value and adjusted *P*-value for this difference.

Table S6. Expression of E2F targets and Hypoxia gene sets

Gene set enrichment analysis (GSEA) of the Hallmark gene set of E2F targets and Hypoxia gene sets. Sheet 1: Read me. Sheet 2: GSEA of E2F target genes in the comparison of transcriptomes of human DS and euploid fetal hearts, identifying genes in the leading edge. Sheet 3: GSEA of E2F target genes in the comparison of transcriptomes of Dp1Tyb and WT mouse embryonic hearts, identifying genes in the leading edge. Sheet 4: GSEA of E2F target genes in the comparison of transcriptomes of Dp1Tyb*Dyrk1a*$^{+/+/-}$ and WT embryonic hearts, identifying genes in the leading edge. Sheet 5: Comparison of the leading genes in the GSEA of E2F target genes of human DS v euploid and mouse Dp1Tyb v WT embryonic hearts. Sheet 6: GSEA of Hypoxia genes in the comparison of transcriptomes of Dp1Tyb and WT embryonic hearts.

Table S7. RNAseq analysis of Dp1Tyb*Dyrk1a*$^{+/+/-}$ E13.5 mouse embryonic hearts.

Sheet 1: Read me. Sheet 2: Expression of all genes in all samples in transcripts per million reads (TPM), and mean expression in each of the two genotypes (Dp1Tyb*Dyrk1a*$^{+/+/-}$ and WT). Sheet 3: Differential gene expression analysis from DESeq2 showing for each gene its location, mean expression across all samples, log2(fold change) between Dp1Tyb*Dyrk1a*$^{+/+/-}$ and WT and the *P*-value and adjusted *P*-value for this difference.

Table S8. Proteomic analysis of Dp1Tyb*Dyrk1a*$^{+/+/-}$ E13.5 mouse embryonic hearts.

Sheet 1: Read me. Sheet 2: Abundance of phospho-peptides in all samples, the Welch difference, a measure of fold-change between Dp1Tyb*Dyrk1a*$^{+/+/-}$ and Dp1Tyb hearts (log2[fold change of the geometric means]), and the *P*-value. Sheet 3: Abundance of peptides in all samples, the log2(fold change) between Dp1Tyb*Dyrk1a*$^{+/+/-}$ and Dp1Tyb hearts, and the FDR-adjusted *P*-value.

Table S9. Effect of Dyrk1a dosage on differential gene expression.

Sheet 1: Read me. Sheet 2: DESeq2 analysis of the transcriptomes of Dp1Tyb v WT embryonic hearts. Sheet 3: DESeq2 analysis of the transcriptomes of Dp1Tyb v Dp1Tyb*Dyrk1a*$^{+/+/-}$ embryonic hearts. Sheet 4: Differentially expressed genes in common between those identified in sheets 2 and 3. Sheet 5: STRING analysis of the common upregulated genes. Sheet 6: STRING analysis of the common downregulated genes. Sheet 7: Genes and proteins upregulated and downregulated in RNAseq or proteomic analysis of Dp1Tyb v Dp1Tyb*Dyrk1a*$^{+/+/-}$ embryonic hearts, indicating genes/proteins in common between the two analyses.

Table S10. RNAseq of Dp1Tyb and WT embryonic hearts from pregnant mice treated with Leucettinib-21 or iso-Leucettinib-21.

Sheet 1: Read me. Sheet 2: Normalized counts of all genes in all samples, and mean expression for each condition. Sheet 3: Differential gene expression analysis from DESeq2 showing for each gene its location, mean expression across all samples, log2(fold change) between Dp1Tyb embryonic hearts treated with iso-Leucettinib-21 and WT embryonic hearts treated with iso-Leucettinib-21, the *P*-value and an indication if the adjusted *P*-value reached significance. Sheet 4: Differential gene expression analysis

from DESeq2 showing for each gene its location, mean expression across all samples, log2(fold change) between Dp1Tyb embryonic hearts treated with Leucettinib-21 and WT embryonic hearts treated with iso-Leucettinib-21, the *P*-value and an indication if the adjusted *P*-value reached significance.
